# Characterization of Hailey-Hailey Disease-mutants in presence and absence of wild type SPCA1 using *Saccharomyces cerevisiae* as model organism

**DOI:** 10.1038/s41598-019-48866-y

**Published:** 2019-08-27

**Authors:** Daniel Muncanovic, Mette Heberg Justesen, Sarah Spruce Preisler, Per Amstrup Pedersen

**Affiliations:** Department of Biology, August Krogh Building, University of Copenhagen, Universitetsparken 13, 2100 Copenhagen, OE Denmark

**Keywords:** Mechanisms of disease, Protein folding

## Abstract

Hailey-Hailey disease is an autosomal genetic disease caused by mutations in one of the two *ATP2C1* alleles encoding the secretory pathway Ca^2+^/Mn^2+^-ATPase, hSPCA1. The disease almost exclusively affects epidermis, where it mainly results in acantholysis of the suprabasal layers. The etiology of the disease is complex and not well understood. We applied a yeast based complementation system to characterize fourteen disease-causing *ATP2C1* missense mutations in presence or absence of wild type *ATP2C1* or *ATP2A2*, encoding SERCA2. In our yeast model system, mutations in *ATP2C1* affected Mn^2+^ transport more than Ca^2+^ transport as twelve out of fourteen mutations were unable to complement Mn^2+^ sensitivity while thirteen out of fourteen to some extent complemented the high Ca^2+^requirement. Nine out of fourteen mutations conferred a cold sensitive complementation capacity. In absence of a wild type *ATP2C1* allele, twelve out of fourteen mutations induced an unfolded protein response indicating that *in vivo* folding of hSPCA1 is sensitive to disease causing amino acid substitutions and four of the fourteen mutations caused the hSPCA1 protein to accumulate in the vacuolar membrane. Co-expression of either wild type *ATP2C1* or *ATP2A2* prevented induction of the unfolded protein response and hSPCA1 mis-localization.

## Introduction

Hailey-Hailey disease (HHD), also known as familial benign chronic pemphigus, is a rare, dominantly inherited, autosomal skin-disorder, mainly characterized by loss of cohesion (acantholysis) between keratinocytes in the suprabasal layers of the skin. Mutations in the *ATP2C1* gene, encoding the Secretory Pathway Ca^2+^/Mn^2+^-ATPase Type 1 (hSPCA1) are known to cause HHD^1,2^ but the underlying mechanism of the disease is far from fully understood. The various model systems that have been used to study the molecular cause of HHD have revealed a disease with great complexity^3–6^.

Despite that currently more than 180 mutations in *ATP2C1* have been deposited in the Human Gene Mutation Database Professional (www.hgmd.cf.ac.uk/ac/index.php), and more than 170 have been listed in recent publications^7,8^ only few have been functionally characterized^9^. The amino acid substitutions causing HHD do not cluster in specific parts of the hSPCA1 primary structure.

hSPCA1 belongs to the family of P-type ATPases - a group of transmembrane proteins that uses the energy in ATP to transport one or more substances across the membrane, in a process involving transient phosphorylation of a conserved aspartate residue^10^.

Four different splice variants of the human hSPCA1, named hSPCA1a-d have been experimentally verified^11^. These hSPCA1 isoforms only differ in their carboxyl-termini. The shortest isoform, hSPCA1c has been shown to be nonfunctional, but the remaining hSPCA1a, -b and –d isoforms all have similar kinetic properties^12^. The precise functional differences among these three variants are still unclear but carboxyl termini of other P-type ATPases are known to play a regulatory role^13–15^.

hSPCA1 localizes to Golgi membranes, particularly *trans*-Golgi^16^, where it transports Ca^2+^ and Mn^2+^ ions into the Golgi lumen^11,17^. *ATP2C1* is ubiquitously expressed in mammalian cells but expression is particularly high in epidermal keratinocytes and varies among other human tissues^2,18,19^. hSPCA1 accumulation has been shown to be markedly reduced in keratinocytes from HHD patients^16^. While *ATP2C1* mutations are disease causing, the complete etiology of the disease is apparently more complex, as only parts of the skin are affected and most patients are symptom-free for the first 2–4 decades of life. It is currently not known how mutations in *ATP2C1* lead to the clinical phenotype and why mainly skin is affected. It is generally believed that the reduced ability to pump Ca^2+^ into Golgi, resulting from haploinsufficiency, is the main cause of HHD^20–22^. The suggestion that a perturbed cellular Ca^2+^ homeostasis is at the root of HHD is further supported by the fact that a Ca^2+^ has several important roles in the skin^2,16,23–29^.

The *Saccharomyces cerevisiae* hSPCA1 orthologue Pmr1 was the first secretory pathway Ca^2+^-ATPase to be identified and characterized^30–32^. Consequently, most of the knowledge we have on SPCA comes from studies of yeast Pmr1. Like its human counterpart, Pmr1 localizes mainly to Golgi but it is also present in low amounts in the ER membrane^17,31–34^. Like hSPCA1, Pmr1 transports Ca^2+^ and Mn^2+^ ions across the ER/Golgi membrane^33^, and one of its functions is to supply ER and Golgi resident enzymes such as glycosyltransferases, kinases and proteases with Mn^2+^ or Ca^2+^^7,30,31,33,35–38^. The requirement for Ca^2+^ ions in ER/Golgi may explain why *pmr1*Δ yeast cells are sensitive to very low extracellular Ca^2+^ concentrations^33,39,40^. *pmr1*Δ yeast cells are also hypersensitive to elevated extracellular Mn^2+^ concentrations^17,41,42^ as they cannot clear Mn^2+^ ions from the cytoplasm, which is essential, as an elevated Mn^2+^ concentration in the cytoplasm is toxic^43–48^.

Human SPCA1 shows 49% homology with yeast Pmr1 and has previously been shown to fully complement the high Ca^2+^ requirement and the Mn^2+^ sensitivity of *pmr1*Δ yeast^17^. Yeast is a unique model organism for studying hSPCA1 mutations as it is currently the only background-free system that allows all endogenous intracellular Ca^2+^- and Mn^2+^ ATPases (*PMR1* and *PMC1*) to be knocked out. Since yeast also lacks other secretory pathway Ca^2+^ pumps (i.e. SERCA-type Ca^2+^ ATPases) and Mn^2+^ pumps, potential interferences from these pumps are absent, too. Loss of both SPCA1 alleles in humans has only been described in very few cases^49,50^ and only as postzygotic, segmental loss of the healthy allele, with a more severe phenotype in the affected areas, indicating that SPCA1 is most likely essential. This is further corroborated by the fact that homozygous knock-out of SPCA1 or SERCA2 in mouse is lethal in the embryonal stage^22,51^. Taken together this indicates that a background-free mammalian model system may be unachievable.

Proper functionality of a protein is dependent on the specific three dimensional structure of the polypeptide chain. Interference with protein folding in the ER, e.g. due to amino acid substitutions, prevention of disulphide bond formation or inhibition of glycosylation^52^, induces an evolutionary conserved and thoroughly investigated signal transduction pathway designated the unfolded protein response (UPR)^53^. In mammalian cells UPR can be induced by three different pathways, mediated by three different sensor proteins: PERK, ATF6 and IRE1. The IRE1 pathway is highly conserved among eukaryotes and also exists in yeast^53–56^. Thus, proteins that induce UPR in yeast are expected to induce UPR in mammalian cells, too. Accumulation of misfolded and unfolded proteins in the ER can lead to ER stress and prolonged or chronic ER stress can ultimately cause apoptosis^53,57^.

In the present study, we have used *Saccharomyces cerevisiae* as model organism for heterologous expression of wild type (wt) and 14 missense variants of *ATP2C1* identified in diagnosed HHD patients. We chose to investigate the shortest functional isoform, hSPCA1a, as this is the “canonical” isoform and because mutations affecting the function of the shortest form would most likely also affect the longer splice variants as only the C-termini differ among isoforms and no missense mutations affect this region^7^. We investigated how gene copy number affects the *in-vivo* Ca^2+^ and Mn^2+^ transport capacity of HHD mutants including its temperature dependency, intracellular localization and accumulation of hSPCA1a protein, in addition to ER-stress induced by expression of HHD variants. In addition we also investigated the effects of co-expressing wild type *ATP2C1* or *ATP2A2*, encoding SERCA2.

## Results

### Expression of ATP2C1a in *Saccharomyces cerevisiae*

**Figure 1:**
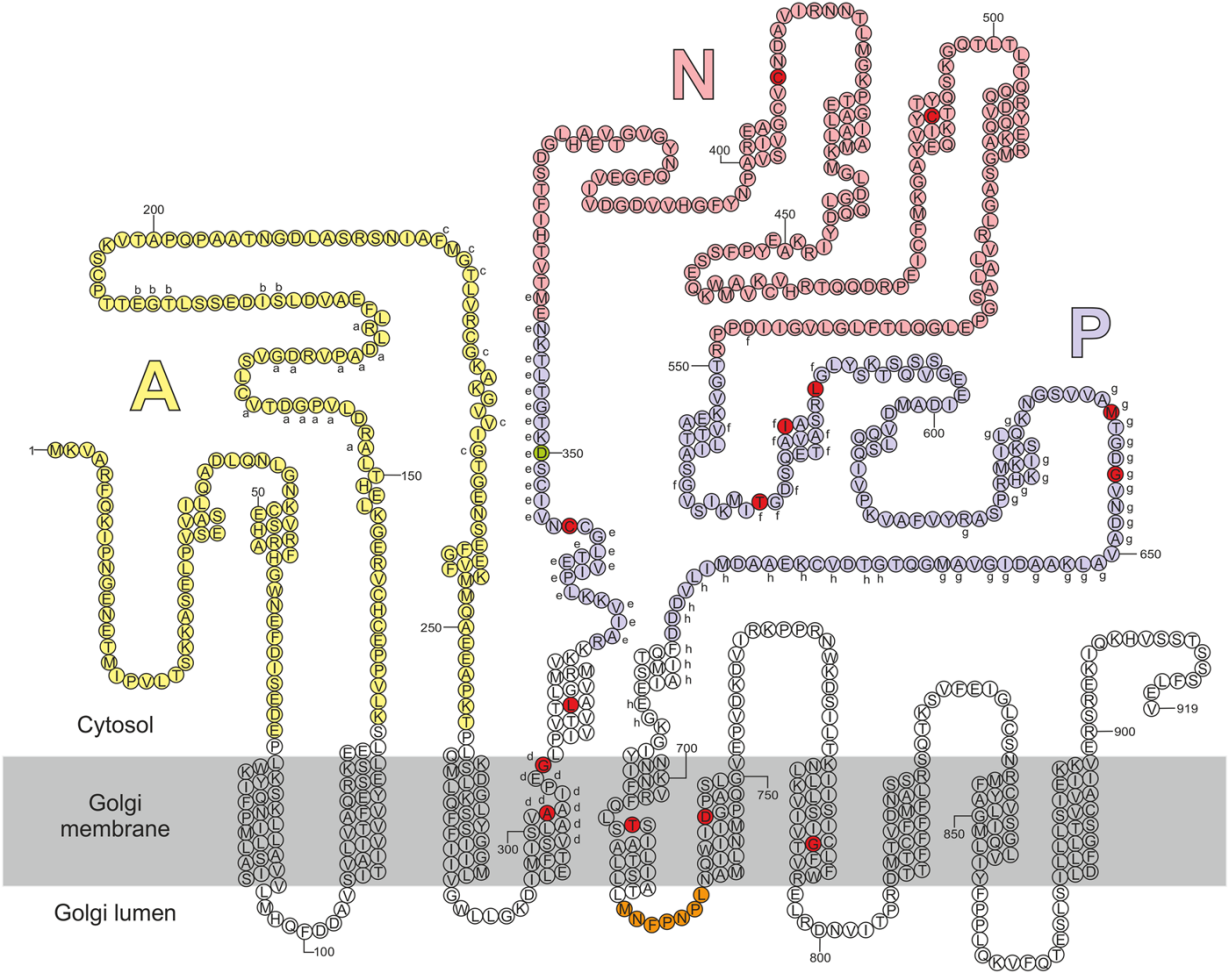
Predicted secondary structure of the hSPCA1a protein. Circles indicate amino acids. The A, N and P domains are colored in light shades of yellow, pink and blue respectively. Conserved amino acids are indicated with lower-case letters (**a**–**h**) corresponding to the eight conserved stretches found in P-type ATPases^103^. The invariant Asp-residue (D350) which is phosphorylated during the reaction cycle is indicated with green. Mutations used in the present study are indicated with dark red. Orange stretch in luminal loop no. 3 indicates the potential BiP-binding hepta-peptide motif. Helix prediction was made using the PSIPRED tool from the Bioinformatics group at University College London (bioinf.cs.ucl.ac.uk/psipred/). hSPCA1a sequence was obtained from UniProt (www.uniprot.org/uniprot/), accession no.: P98194.

To address to what extent *ATP2C1a* gene dosage, temperature, protein accumulation, protein localization and interference with *in vivo* hSPCA1a protein folding may contribute to the etiology of HHD we selected fourteen disease-causing amino acid substitutions located in various domains of the hSPCA1a primary structure (Fig. 1) and expressed them in the *pmc1*Δ *pmr1*Δ *cnb1*Δ yeast strain PAP4920 or derivatives. As Hailey-Hailey patients usually carry one wt allele and one mutant allele, as well as other Ca^2+^-ATPases we additionally generated yeast strains that co-express either wt *ATP2C1a* (encoding hSPCA1a) or wt *ATP2A2b* (encoding hSERCA2b) and each of the investigated mutant alleles. The PAP4920 strain was selected because it is presently the only eukaryotic cell model without endogenous Ca^2+^ and Mn^2+^-ATPase activity and it is equipped with a chromosomally integrated unfolded protein response reporter^58^ for monitoring *in vivo* protein folding problems in the ER due to hSPCA1a expression.

### Most HHD-associated *ATP2C1a* variants affect *in vivo* Ca^2+^ transport in a copy number dependent way

**Figure 2:**
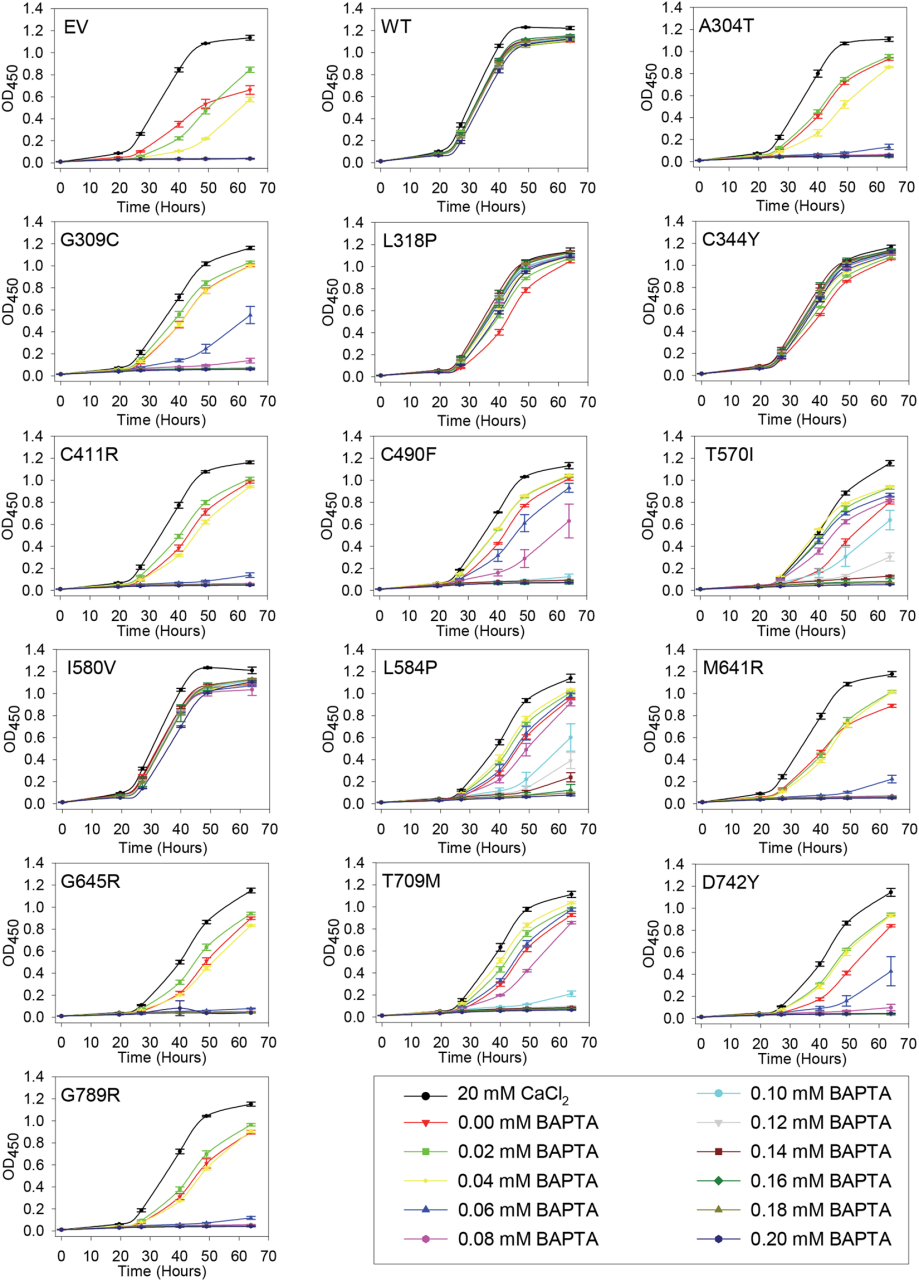
Complementation of the calcium dependent phenotype of the *pmr1*Δ *pmc1*Δ *cnb1*Δ yeast host strain by integrative hSPCA1a mutants. Yeast strain PAP4920 expressing no hSPCA1a (Empty Vector, EV), wt hSPCA1a (WT) or the indicated HHD hSPCA1a variants from the integrative vector (pPAP5480, Supplementary Fig. S1) were grown at 30 °C in 96-well plates in liquid media with galactose as carbon source supplemented with 20 mM CaCl_2_ or BAPTA concentrations ranging from 0 to 0.2 mM in increments of 0.02 mM as indicated. Each culture was grown in triplicates and the average OD_450_ at the given time was plotted with respect to time of inoculation. Growth media containing BAPTA (including 0 mM BAPTA) do not have any added Ca^2+^.

**Figure 3:**
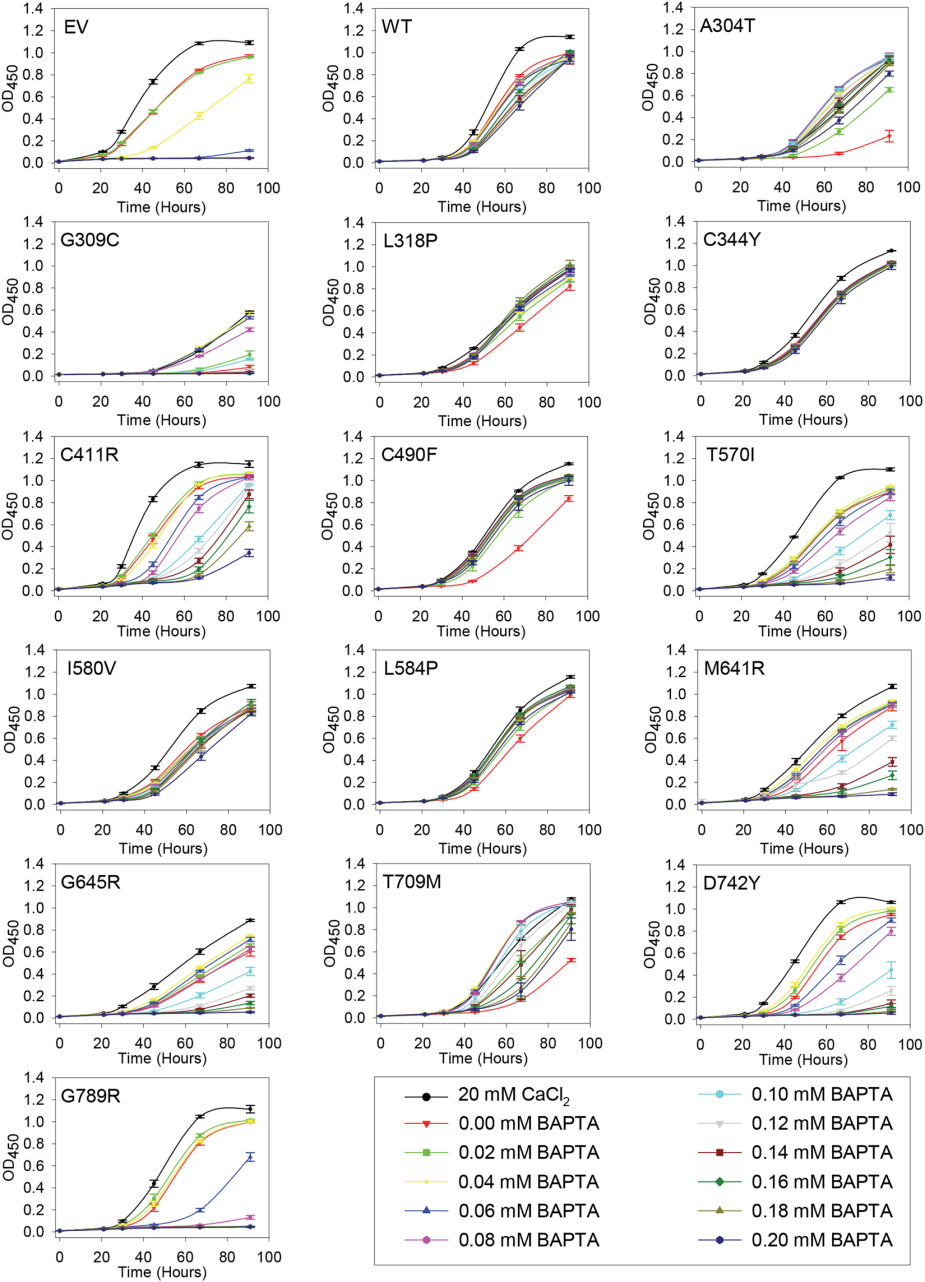
Complementation of the calcium dependent phenotype of the *pmr1*Δ *pmc1*Δ *cnb1*Δ yeast host strain by replicative hSPCA1a mutants. Yeast strain PAP4920 expressing no hSPCA1a (Empty Vector, EV), wt hSPCA1a (WT) or the indicated HHD hSPCA1a variants from the replicative vector (pPAP4997, Supplementary Fig. S1) were grown at 30 °C in 96-well plates in liquid media with galactose as carbon source supplemented with 20 mM CaCl_2_ or BAPTA concentrations ranging from 0 to 0.2 mM in increments of 0.02 mM as indicated. Each culture was grown in triplicates and the average OD_450_ at the given time was plotted with respect to time of inoculation. Growth media containing BAPTA (including 0 mM BAPTA) do not have any added Ca^2+^.

Increasing the CaCl_2_ concentration in the growth media from the standard 0.4 µM^59^ to 20 mM^60^ results in a concentration high enough to supply intracellular organelles with sufficient Ca^2+^ in the *pmc1*Δ *pmr1*Δ *cnb1*Δ yeast strain^61^. Requirement for a high extracellular Ca^2+^ concentration can be complemented by human wt *ATP2C1a*^17^. To obtain detailed information on how complementation depends on *ATP2C1a* gene dosage, yeast cells producing wt hSPCA1a or HHD variants from either a single cDNA copy (integrative) or approximately twenty copies (replicative, Supplementary Fig. S1) were grown in 96-well microplates at 30 °C in twelve different media, each having a unique Ca^2+^ concentration (Figs 2 and 3). The growth profiles obtained from this microplate assay reflect how efficient each hSPCA1a variant is at distributing cytosolic Ca^2+^ into the secretory pathway.

Data in Figs 2 and 3 show that yeast cells carrying either the chromosomally integrated or the replicative empty expression vector sustained growth at BAPTA concentrations up to 0.04 mM, while growth of yeast cells expressing wt *ATP2C1a* from either a single cDNA copy or from twenty copies was hardly affected by the applied BAPTA concentrations. Irrespective of copy number amino acid substitutions L318P, C344Y and I580V showed a BAPTA dependent growth profile similar to wt. In contrast 10 out of 14 mutants showed a copy number dependent complementation capacity as A304T, G309C, C411R, C490F, T570I, L584P, M641R, G645R, T709M and D742Y sustained growth no better or only marginally better than the empty vector when expressed from the integrative plasmid, but at a copy number of twenty A304T, C490F and L584P showed BAPTA dependent growth profiles similar to wt. The complementation capacity of the other seven variants (G309C, C411R, T570I, M641R, G645R, T709M and D742Y) was significantly better than the empty vector but poorer than wt. HHD variant G789R stands out as it did not confer any BAPTA tolerance greater than that of the empty vector when expressed from a single cDNA copy and was only marginally better than empty vector when expressed from the multi copy plasmid. The BAPTA related phenotypes of all HHD variant are summarized in Table 1.

**Table 1:** Phenotypes of homozygous *ATP2C1* alleles.

	BAPTA resistance^a^ (*liquid*) (solid)				Mn^2+^ resistance^a^ (*liquid*) (solid)				Ca^2+^ sensitivity^b^ (*liquid*) (solid)				Temperature sensitivity^c^		Protein accumulation^d^	Protein localization	UPR^e^
Copy Number ^f^	LC		HC		LC		HC		LC		HC		LC	HC	HC	HC	
EV	*0*	**0**	*0*	**0**	*0*	**0**	*0*	**0**	*0*	**0**	*0*	**0**	**0**	**0**	0	−	Low
WT	++	++	++	++	++	**++**	++	**++**	*0*	**0**	*0*	**0**	**HS**	**HS**	High	Golgi	Low
A304T	*0*	**0**	++	+	*0*	**0**	*0*	**0**	*0*	**0**	+	**0**	**0**	**0**	High	Golgi	High
G309C	*0*	**0**	+	**+**	*0*	**0**	*0*	**0**	*0*	**0**	+	**+**	**CS**	**CS**	High	Vacuole	Low
L318P	++	++	*++*	**++**	*0*	**0**	*0*	**0**	*+*	**+**	+	**+**	**CS**	**CS**	High	Golgi	High
C344Y	++	++	*++*	**++**	*0*	**0**	*+*	**+**	*+*	**0**	*0*	**0**	**CS**	**CS**	Low	Golgi	High
C411R	*0*	**0**	+	**+**	*0*	**0**	*0*	**0**	*0*	**0**	*0*	**+**	**CS**	**CS**	Low	Golgi	High
C490F	*0*	**0**	++	**+**	*0*	**0**	*0*	**0**	*0*	**0**	+	**+**	**0**	**HS**	Low	Golgi	High
T570I	+	**0**	+	**+**	*0*	**0**	*0*	**0**	*+*	**0**	*0*	**+**	**HS**	**0**	Low	Golgi	High
I580V	++	**++**	++	**++**	++	**++**	++	++	*0*	**0**	*0*	**0**	**HS**	**HS**	High	Vacuole	High
L584P	+	**+**	*++*	**++**	*0*	**0**	*0*	**0**	*0*	**0**	+	**0**	**CS**	**CS**	Low	Golgi	High
M641R	*0*	**0**	+	**+**	*0*	**0**	*0*	**0**	*0*	**0**	+	**+**	**CS**	**CS**	Low	Golgi	High
G645R	*0*	**0**	+	**+**	*0*	**0**	*0*	**0**	*0*	**0**	+	**+**	**0**	**CS**	High	Vacuole	High
T709M	*+*	**0**	+	**+**	*0*	**0**	*0*	**0**	*0*	**0**	+	**+**	**CS**	**CS**	High	Golgi	Low
D742Y	*0*	**0**	+	**+**	*0*	**0**	*0*	**0**	*0*	**0**	*0*	**+**	**CS**	**CS**	High	Vacuole	High
G789R	*0*	**0**	*0*	**0**	*0*	**0**	*0*	**0**	*0*	**0**	*0*	**0**	**0**	**0**	Low	Golgi	High
Pmr1	*ND*	**ND**	*ND*	**ND**	*ND*	**ND**	*ND*	**ND**	*ND*	**ND**	*ND*	**ND**	**ND**	**ND**	Low	Golgi	Low
Pmc1	*ND*	**ND**	*ND*	**ND**	*ND*	**ND**	*ND*	**ND**	*ND*	**ND**	*ND*	**ND**	**ND**	**ND**	High	Vacuole	Low

^a^BAPTA resistance and Mn^2+^ resistance were based on data in Figs 2–6 (30 °C only) and given a value of 0 if complementation was empty vector like, “+” if growth was intermediary and “++” if growth was wt-like. ^b^Ca^2+^ sensitivity was determined from Figs 2, 3 and 6 (30 °C only) and given a value of 0 if no Ca^2+^ dependent inhibition was observed or a “+” if growth was inhibited at media containing 20 mM CaCl_2_ or 0 mM BAPTA.^c^Temperature sensitivity was categorized from the spot assay in Fig. 6, as being either Cold Sensitive (CS), Heat Sensitive (HS) or temperature independent (0). ^d^Protein accumulation was based on Fig. 8 and given two values: High if protein accumulation was wt-like, Low if protein accumulation was lower than wt. ^e^UPR data from Fig. 14 were given two values: High if the induced UPR level was at least two times greater than that of wt and Low if the induced UPR level was less than two times that of wt. ^e^Copy Number indicates the expression from the integrative (Low Copy, LC) or from the replicative (High Copy, HC) plasmids. *Liquid*, cells were grown in liquid medium in microplates; **solid**, cells were spotted on solid agar medium; ND, not determined.

### Most HHD mutations show copy number independent Mn^2+^ sensitivity

**Figure 4:**
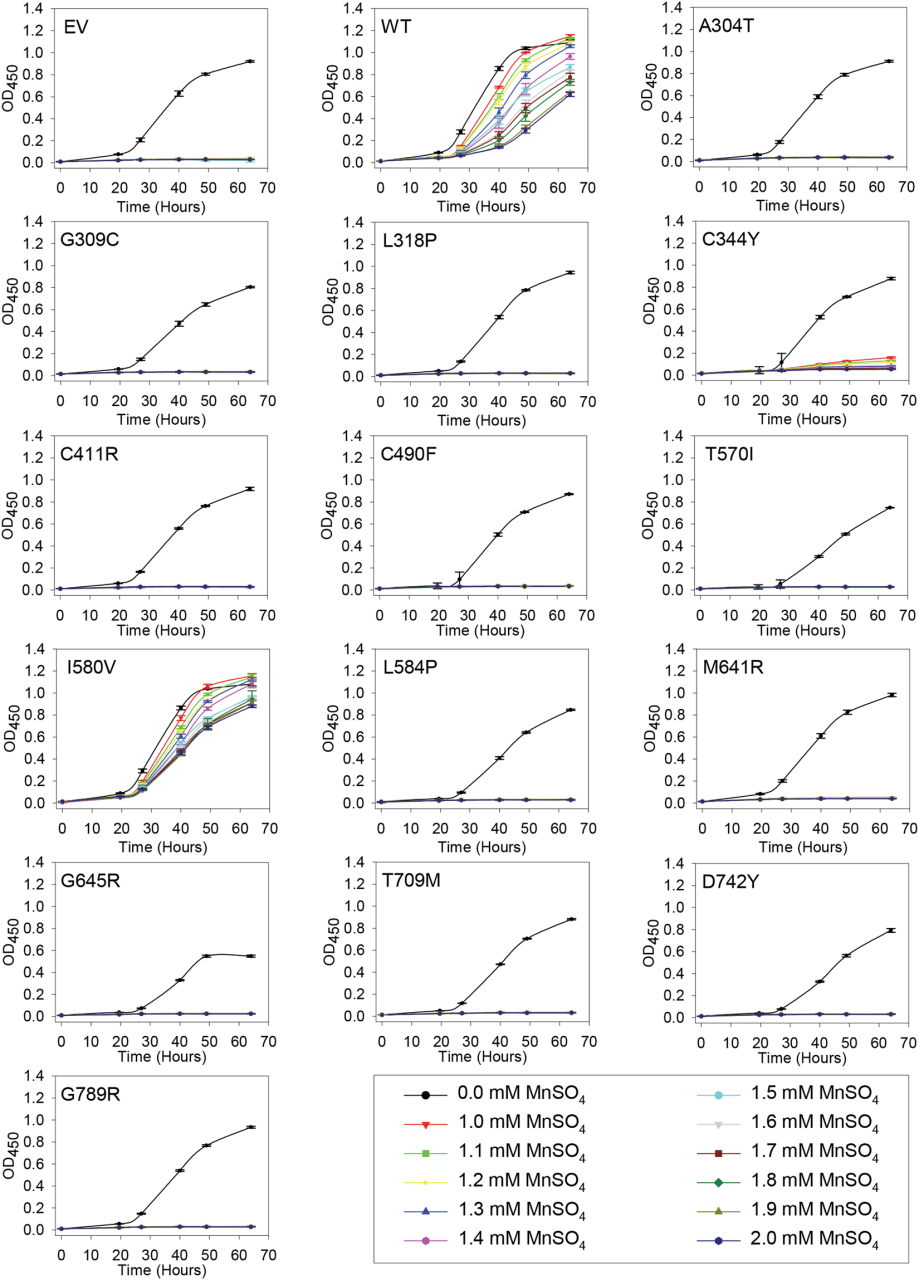
Complementation of the manganese sensitive phenotype of the *pmr1*Δ *pmc1*Δ *cnb1*Δ yeast host strain by integrative hSPCA1a mutants. Yeast strain PAP4920 expressing no hSPCA1a (Empty Vector, EV), wt hSPCA1a (WT) or the indicated HHD hSPCA1a variants from the integrative vector (pPAP5480, Supplementary Fig. S1) were grown at 30 °C in 96-well plates in liquid media with galactose as carbon source supplemented with 20 mM CaCl_2_, and MnSO_4_ ranging from 0 to 2.0 mM in increments of 0.1 mM as indicated. Each culture was grown at 30 °C, in triplicates, and average OD_450_ was plotted with respect to time of inoculation.

**Figure 5:**
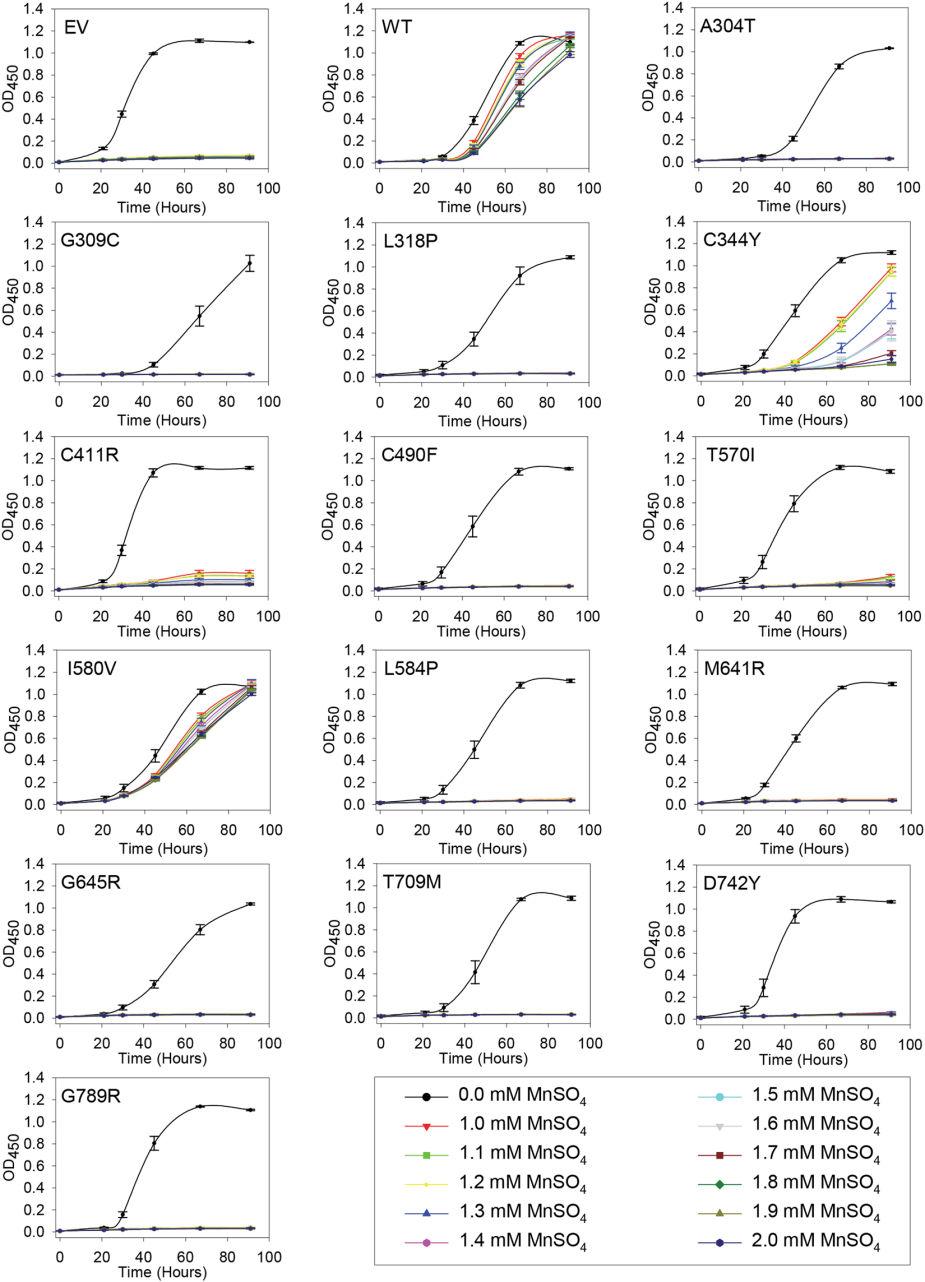
Complementation of the manganese sensitive phenotype of the *pmr1*Δ *pmc1*Δ *cnb1*Δ yeast host strain by replicative hSPCA1a mutants. Yeast strain PAP4920 expressing no hSPCA1a (Empty Vector, EV), wt hSPCA1a (WT) or the indicated HHD hSPCA1a variants from the integrative vector (pPAP5480, Supplementary Fig. S1) were grown at 30 °C in 96-well plates in liquid media with galactose as carbon source supplemented with 20 mM CaCl_2_, and MnSO_4_ ranging from 0 to 2.0 mM in increments of 0.1 mM as indicated. Each culture was grown at 30 °C, in triplicates, and average OD_450_ was plotted with respect to time of inoculation.

Growth of *pmc1*Δ *pmr1*Δ *cnb1*Δ yeast is very sensitive to Mn^2+^ as absence of Pmr1 abolishes removal of toxic amounts of Mn^2+^ accumulating in the cytosol^17^. To obtain detailed information on how *ATP2C1a* gene dosage influences complementation of the Mn^2+^ sensitive phenotype, yeast cells producing wt hSPCA1a or HHD variants from either a single cDNA copy or approximately twenty copies were grown in 96-well microplates at 30 °C in 12 different growth media (Figs 4 and 5). Mn^2+^ concentrations between 1.0 mM and 2.0 mM MnSO_4_ were investigated because yeast expressing wt hSPCA1a showed differential growth in this interval and 1.0 mM MnSO_4_ was sufficient to prevent growth of cells carrying the empty vector.

Although growth of yeast cells expressing wt *ATP2C1a* from both a single cDNA copy and approximately twenty copies was sensitive to Mn^2+^, growth was sustained up till 2.0 mM Mn^2+^ (Figs 4 and 5). Of the fourteen investigated HHD variants only I580V conferred a copy number independent Mn^2+^ resistance on par with wt, while C344Y showed some Mn^2+^ resistance and only when expressed from the replicative plasmid. The remaining 12 variants were all unable to rescue the Mn^2+^ sensitive phenotype at any copy number. The Mn^2+^ sensitivity is summarized in Table 1.

### Amino acid substitutions in HHD preferentially interfere with *in vivo* Mn^2+^ transport

As summarized in Table 1 a majority of the investigated HHD variants (A304T, G309C, C411R, C490F, M641R, G645R, D742Y and G789R) neither complemented the Mn^2+^ toxicity nor the BAPTA sensitivity better than the empty vector when expressed from a single cDNA copy (Figs 2 and 4). HHD variants T570I, L584P and T709M showed Ca^2+^ transport only marginally better than the empty vector but no Mn^2+^ transport when expressed from a single cDNA copy. HHD variants L318P, C344Y and I580V stood out, as the two former showed wt like Ca^2+^ transport capacity but were devoid of any Mn^2+^ transport capacity, while I580V complemented BAPTA sensitive growth and Mn^2+^ toxicity equally well as wt.

When expressed from the replicative vector, all HHD variants except G789R were to some extent able to complement the BAPTA-sensitive growth phenotype of the *pmc1*Δ *pmr1*Δ *cnb1*Δ strain (Fig. 3), whereas only C344Y and I580V were able to sustain growth on Mn^2+^ media (Fig. 5). HHD variants L318P, C344Y, C490F, I580V and L584P showed a wt-, or near wt-like phenotype in presence of BAPTA. Three of the five mutations showed no Mn^2+^ transport, only I580V showed a wt-like Mn^2+^ resistant growth phenotype and C344Y showed partial Mn^2+^ resistance. Substitutions T570I, T709M and D742Y also showed considerably BAPTA resistance but no Mn^2+^ resistance while G789R showed no Ca^2+^ transport and no Mn^2+^ transport. I580V was the only one to show a copy number independent wt phenotype with respect to both Ca^2+^ and Mn^2+^ transport.

Compared to wt and single *pmr1* knockout strains a *pmc1*Δ *pmr1*Δ yeast strain shows substantially increased influx of Ca^2+^ and elevated Ca^2+^ accumulation when grown in low Ca^2+^ media^6^. Increased cytosolic Ca^2+^ activates the serine/threonine phosphatase calcineurin^6,62^ causing dephosphorylation and potential subsequent inhibition of Cch1, a subunit of the channel that forms the high affinity Ca^2+^-uptake system (HACS)^61–63^. Since our yeast model system lacks *PMR1*, *PMC1* and *CNB1*, Ca^2+^ may hyper-accumulate in the cytoplasm and generate a large concentration gradient across the membranes of ER/Golgi. Thus, even a poorly functioning Ca^2+^-ATPase may appear to have a significant Ca^2+^-transport activity. This could severely influence the observed difference in Ca^2+^ and Mn^2+^ transport of the investigated HHD variants (Figs 2–5). As most of the investigated HHD-hSPCA1a variants displayed some degree of Ca^2+^ transporting activity, we tested the BAPTA and Mn^2+^ tolerance of the investigated hSPCA1a variants expressed from the replicative plasmid in a *pmr1*Δ single knockout strain. The results in Supplementary Figs S2 and S3 show that the BAPTA and Mn^2+^ tolerance conferred by the hSPCA1a variants in the *pmr1*Δ strain is similar to that observed in the triple *pmr1*Δ *pmc1*Δ *cnb1*Δ strain. This suggests that the observed BAPTA and Mn^2+^ tolerance of the tested HHD-SPCA1a variants reflects ATPase function and is not caused by hyper-accumulation of Ca^2+^ in the cytoplasm.

### HHD-mutations do not exert a dominant-negative effect on wt hSPCA1a

HHD patients are heterozygous for the *ATP2C1* gene. To investigate the effects of co-expressing each HHD variant with wt hSPCA1 we analyzed the complementation capacity using the micro plate growth assay. As our results with homozygous hSPCA1a expression showed gene dosage dependent complementation (Figs 2–5), we co-expressed wt *ATP2C1a* and each of the HHD-*ATP2C1a* variants from a single cDNA copy (heterozygous, integrative, Supplementary Figs S4 and S5) and wt *ATP2C1a* and each of the HHD- *ATP2C1a* variants from multi copy plasmids (heterozygous, replicative, Supplementary Figs S6 and S7).

The results in Supplementary Figs S4–S7 show that presence of a wt *ATP2C1a* rescued both the BAPTA and the MnSO_4_ sensitive growth phenotypes observed in hSPCA1a homozygous yeast.

As apparent from Supplementary Figs S4–S7 we did not observe any dominant-negative effect of HHD-mutations on wt hSPCA1a.

### Several HHD-variants display sensitivity to Ca^2+^

**Figure 6:**
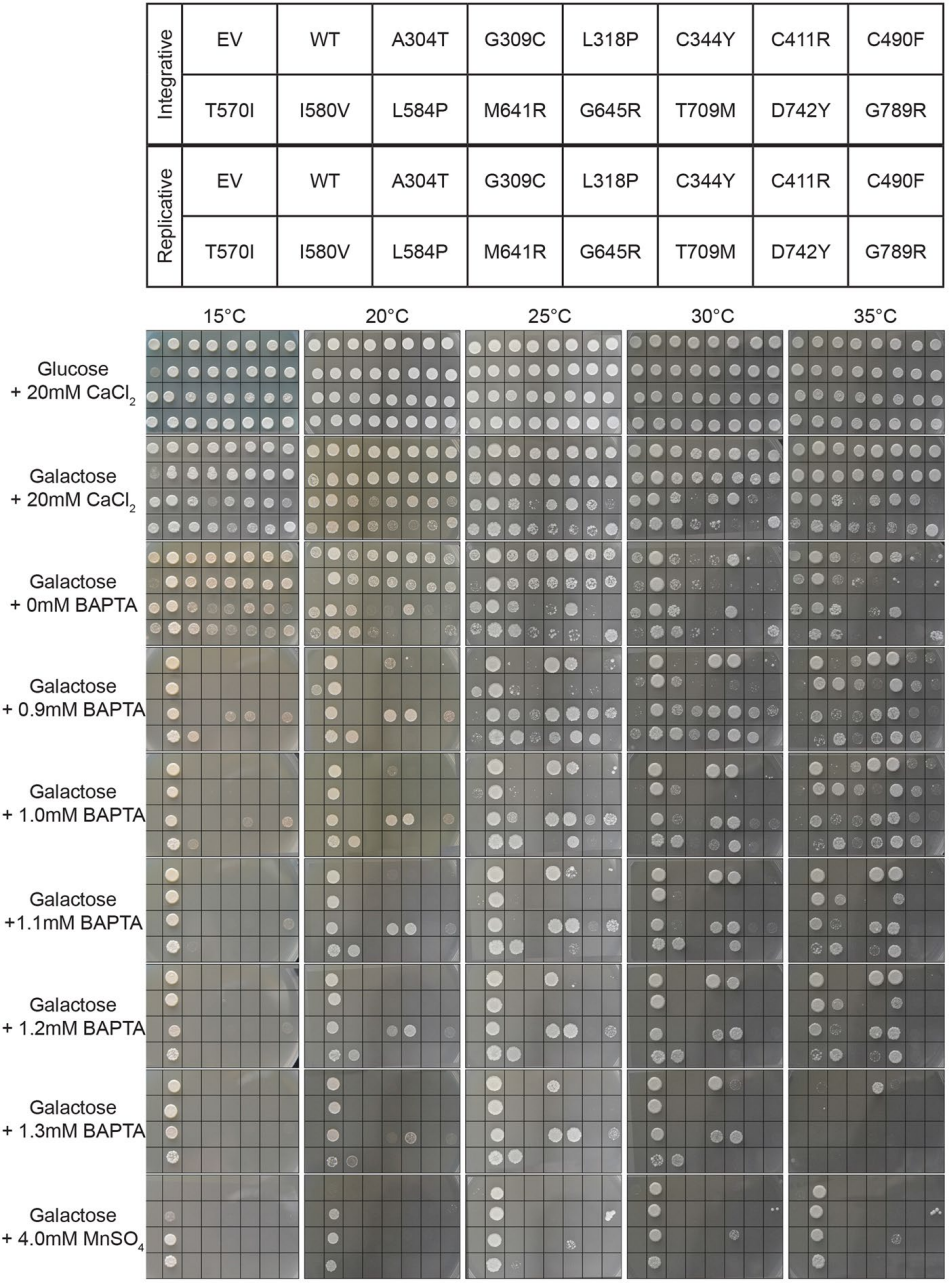
Temperature and copy number dependent complementation by HHD-mutant hSPCA1a. Yeast strain PAP4920 (*pmc1*Δ *pmr1*Δ *cnb1*Δ) expressing no hSPCA1a (empty vector, EV), wt hSPCA1a (WT) or the indicated hSPCA1a HHD variants from either the integrative plasmid pPAP5480 (copy number of one, rows 1 and 2 in each picture) or the replicative plasmid pPAP4997 (copy number of 20, rows 3 and 4 in each picture) as indicated in the top pane, were spotted on agar-plates containing the media shown on the left of the lower pane. All media are synthetic defined media containing either glucose or galactose as the only carbon source as indicated. Media containing BAPTA (including 0 mM BAPTA) did not have any added CaCl_2_. Plates were incubated at 15 °C, 20 °C, 25 °C, 30 °C or 35 °C as indicated, and photographed on a daily basis. Due to temperature dependent growth rates the selected pictures in panels Glucose +20 mM CaCl_2_, Galactose +20 mM CaCl_2_ and Galactose +0 mM BAPTA were taken at the earliest time points where yeast expressing wt *ATP2C1a* and yeast cells expressing no *ATP2C1a* had generated a clearly visible spot. The rest of the pictures were taken at time points where yeast cells expressing wt *ATP2C1a* had generated a visible spot and yeast cells expressing no *ATP2C1a* had failed to generate a spot. Results of one out of two experiments are shown.

For each HHD allele we expected a positive correlation between Ca^2+^ availability in the medium and complementation as we are well below the level where Ca^2+^ becomes toxic^40^. However, many of the investigated HHD-variants display a more complex growth phenotype. In addition to the BAPTA sensitive growth described in a previous paragraph, data in Fig. 6 show that some of the mutants exposed a Ca^2+^-sensitive phenotype. This was observed in liquid media containing galactose and 20 mM CaCl_2_ (Figs 2 and 3), on agar plates containing galactose and 20 mM CaCl_2_ or agar plates containing galactose and 0 mM BAPTA (and no added Ca^2+^) (Fig. 6). The same HHD variants that showed a Ca^2+^-sensitive growth inhibition were at least able to grow on media containing the lowest amount of BAPTA.

To simplify, the growth phenotypes observed on agar plates containing Ca^2+^ or BAPTA at 30 °C from Fig. 6 are summarized in Supplementary Fig. S8. When expressed from the integrative vector at 30 °C on agar plates only L318P exposed a Ca^2+^-sensitive growth phenotype on media containing galactose and 0 mM BAPTA.When expressed from the replicative vector at 30 °C on agar plates, 10 of the 14 investigated HHD-mutants, including G309C, L318P, C411R, C490F, T570I, L584P, M641R, G645R, T709M and D742Y, displayed Ca^2+^-sensitivity in media containing galactose and 20 mM CaCl_2_ or galactose and 0 mM BAPTA. The observed growth inhibition is not simply caused by the presence of an excess amount of Ca^2+^ in the growth medium since all variants, including the empty vector (EV), sustained growth on agar plates containing glucose and 20 mM CaCl_2_ at 30 °C. The inhibition is also not caused by the combination of galactose and 20 mM CaCl_2_ since the negative control (empty vector, EV) could sustain growth at media containing galactose and 20 mM CaCl_2_. The inhibition is thus caused by expression of particular *ATP2C1*a alleles (i.e. the phenotype is restricted to growth on galactose) and the level of expression of the HHD-alleles (as the growth inhibition was much more pronounced for the replicative variants) in combination with the particular concentration of Ca^2+^ in the medium.

A similar inhibition of growth at 0 mM BAPTA was also observed in liquid media (Figs 2 and 3). While inhibition of growth on agar plates containing no added Ca^2+^ and no BAPTA was observed for the majority of the investigated HHD-alleles only HHD-variants L318P and C344Y showed growth inhibition at 0 mM BAPTA when expressed from the integrative vector (Fig. 2, the red curve with triangle symbols is below all other growth curves). Similarly, when expressed from the replicative plasmid, far fewer HHD-variants showed growth inhibition in liquid media, compared to solid media (Fig. 3). HHD-variants A304T, L318P, C490F, L584P and T709M showed a clear inhibition when grown at 0 mM BAPTA. Only three of the 8 HHD-mutants that showed growth inhibition in galactose and 20 mM CaCl_2_ on solid media displayed growth inhibition in galactose and 20 mM CaCl_2_ in liquid media. There is thus no clear correlation between growth inhibition in liquid media and in solid media.

### Cold sensitivity is a common phenotype exposed by HHD-mutants

The growth experiments in Figs 2 and 3 revealed that most HHD-hSPCA1a variants retained some Ca^2+^ transport activity at 30 °C depending on plasmid copy number while Mn^2+^ transport was more affected. To investigate whether temperature effects the ability of the HHD-hSPCA1a variants to complement the BAPTA sensitive and Mn^2+^ sensitive growth phenotypes of the *pmc1*Δ *pmr1*Δ *cnb1*Δ host strain, yeast expressing wt *ATP2C1a*, HHD-*ATP2C1a* or no *ATP2C1a* from either the integrative plasmid or the replicative plasmid (Supplementary Fig. S1), were spotted on agar plates and incubated at 15 °C, 20 °C, 25 °C, 30 °C or 35 °C (Fig. 6).

**Figure 7:**
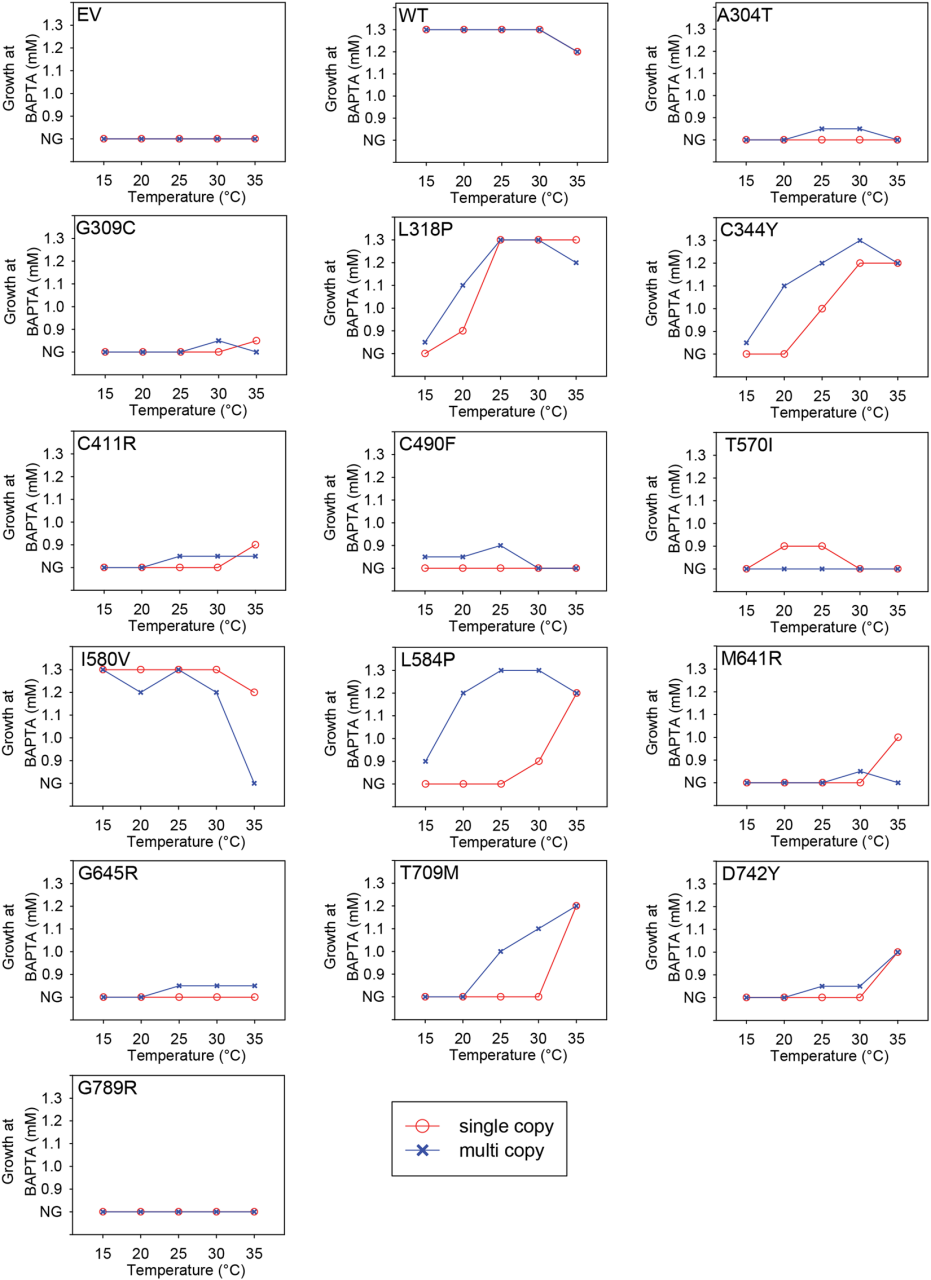
Expression of various *ATP2C1a* alleles confers a temperature dependent complementation profile. The maximal BAPTA concentration allowing generation of a growth spot at 15 °C, 20 °C, 25 °C, 30 °C and 35 °C was determined from the complementation assay in Fig. 6 and plotted with respect to temperature. Blue curves represent expression from the 2 µ based plasmid, pPAP4997, while red curves are based upon expression from the single copy integrative plasmid pPAP5480. Mutants that showed growth at 0.9 mM BAPTA that was considerably better than the empty vector, but still poorer that wt were given a value between no growth (NG) and 0.9 mM BAPTA. The temperature profiles categorized each mutation as either cold sensitive (CS), heat sensitive (HS) or temperature insensitive (TI) when compared to that of wt.

Apart from yeast carrying the empty expression vector, only amino acid substitution G789R abolished functionality at any temperature and BAPTA concentration. The remaining 13 HHD mutants were able to grow to some extent and depending on copy number, at BAPTA concentrations not tolerated by the empty vector-control strain. The complementation capacity for each HHD-*ATP2C1a* variant with respect to temperature is summarized in Fig. 7.

It can be seen from Fig. 7 that relative to wt and irrespective of copy number, HHD-variants L318P, C344Y, L584P, T709M and D742Y conferred a very clear cold sensitive (CS) phenotype, displaying poorer or no BAPTA tolerance at lower temperatures. However, it can be seen from Fig. 6 that complementation by most CS-mutants was observed at a wider range of temperatures when *ATP2C1a* was expressed from the multi copy plasmid compared to expression from the single copy, integrative plasmid. HHD-variants A304T, C411R, M641R and G645R also displayed a CS phenotype, albeit less clear than the HHD-mutations mentioned above. Only substitutions C490F and I580V conferred a heat sensitive phenotype, the former only when expressed from the multi copy plasmid and the latter most pronounced when expressed from the multi copy plasmid. Amino acid substitutions G309C and T570V each showed a more complex phenotype. G309C only complemented weakly at 30 °C and 35 °C at a copy number of twenty and one respectively, indicating a CS-like phenotype; T570I only showed complementation at 20 °C and 25 °C and only after expression from the single copy plasmid, indicating a mixed temperature sensitive phenotype.

Interestingly, the observed Ca^2+^ sensitive phenotype did not display temperature variations among the various HHD-variants, but instead displayed a generally less pronounced Ca^2+^ sensitivity at 15 °C.

The degree of complementation on solid medium is complicated to quantify in contrast to growth in liquid media. To confirm the combined effects of temperature and *ATP2C1a* copy number observed in the spot assays in Fig. 6, we investigated BAPTA sensitive growth at 35 °C (Supplementary Figs S9 and S10) in liquid media in microplates for comparison with growth at 30 °C (Figs 2 and 3). For this analysis we selected representative mutants showing different phenotypes at 30 °C and 35 °C on agar plates (L318P, C344Y, I580V, L584P, M641R, T709M and G789R). In agreement with the temperature profiles in Fig. 7, the microplate results in Fig. 2 and Supplementary Fig. S9 show that after single copy expression wt performs better at 30 °C than at 35 °C and L318P complements equally well at both temperatures and better than wt at 35 °C. C344Y is seen to complement equally well at 30 °C and 35 °C, while I580V is performing considerably better at 30 °C than at 35 °C. In accordance with the plate assay L584P, M641R and T709M each complemented better at 35 °C than at 30 °C in liquid medium. G789R was included as a control as it does not perform better than the empty vector at any of the investigated temperatures. The microplate results in Fig. 3 and Supplementary Fig. S10 from multi-copy expression confirm the spot assays as wt is seen to perform better at 30 °C than at 35 °C, L584P performs better at 30 °C than at 35 °C, while C344Y performs well independent of temperature. The ability of L318P and I580V to complement is considerable reduced at 35 °C compared to 30 °C, while complementation by M641R is marginally reduced at 35 °C. T709M performs better at 35 °C than at 30 °C while G789R was included as a control as it does not complement at any temperature. In agreement with the data in Fig. 6 it can be seen from Figs 2 and 3 that integrative expression of L318P and I580V caused better complementation than the corresponding replicative expression.

### HHD-hSPCA1a protein accumulation is allele specific

**Figure 8:**
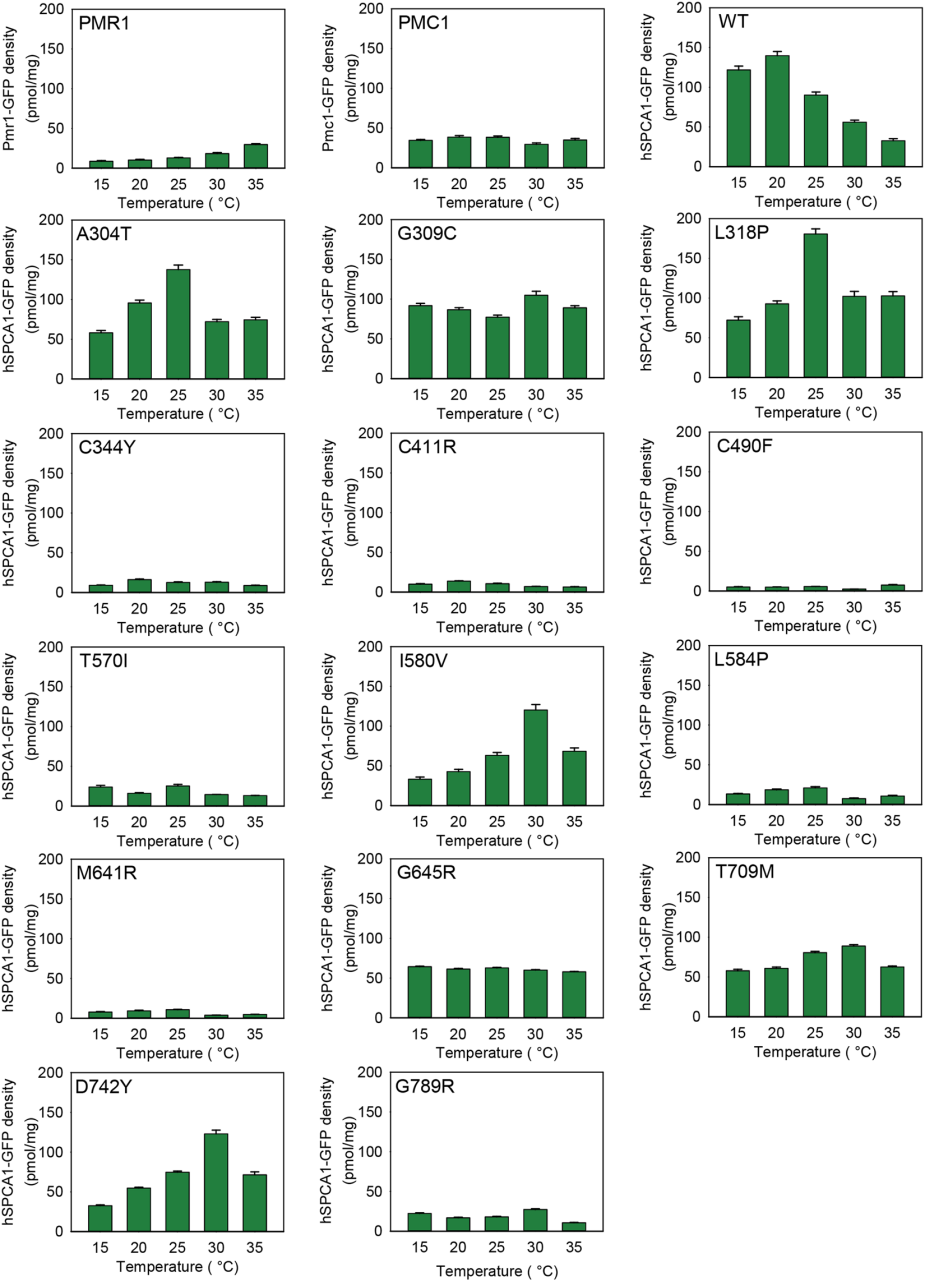
Allele specific accumulation of GFP tagged proteins in yeast membranes expressed at 15 °C, 20 °C, 25 °C, 30 °C and 35 °C. Crude membranes from yeast strain PAP4920 expressing Pmr1-GFP, Pmc1-GFP, wt hSPCA1a-GFP or HHD hSPCA1a-GFP from the replicative vector pPAP4997 were analyzed for fluorescence as described in Materials and Methods. Yeast cultures were inoculated in standard minimal medium supplemented with 20 mM CaCl_2_ in shake flasks at 15 °C, 20 °C, 25 °C, 30 °C or 35 °C at OD_450_ = 0.05. Cultures were harvested at OD_450_ = 1.0–1.5. GFP fluorescence was measured in equal amounts of purified yeast crude membranes containing 25 µg total membrane protein. Fluorescence was converted to pmol per mg crude membranes as described in Materials and Methods. Each meassurment was made in triplicate.

In order to investigate how the combination of HHD mutation and temperature affects hSPCA1a protein accumulation, we C-terminally tagged wt- and the 14 different HHD hSPCA1a variants, as well as the yeast endogenous Ca^2+^ ATPases Pmc1 and Pmr1, with yeast enhanced GFP (yEGFP), and expressed them from the replicative vector at 15 °C, 20 °C, 25 °C, 30 °C and 35 °C in the *pmc1*Δ *pmr1*Δ *cnb1*Δ strain PAP4920. Data in Supplementary Fig. S11 show that presence of the GFP tag does not influence the activity of hSPCA1a. GFP fluorescence emitted from equal amounts of purified yeast membranes therefore reflects membrane accumulation of GFP tagged hSPCA1a protein. Figure 8 shows that Pmc1 and Pmr1 accumulated in a more or less temperature-insensitive way. Wild type hSPCA1a accumulated to a comparable level as the endogenous Ca^2+^ ATPases at 30 °C and 35 °C while at 15 °C, 20 °C and 25 °C, it accumulated to 2–3 times the density observed for Pmc1 and Pmr1. HHD-variants A304T, G309C, L318P, I580V, G645R, T709M and D742Y protein accumulation was comparable to that of the wt hSPCA1a. Of these, G309C, G645R and T709M accumulated hSPCA1a protein in a more or less temperature insensitive manner. A304T and L318P protein accumulation peaked at 25 °C while both I580V and D742Y peaked at 30 °C. The remaining 7 HHD-variants all accumulated protein well below that observed for wt but on par with the Pmr1 orthologue. From the data summarized in Table 1 we conclude that temperature does not have a great effect on protein accumulation in the majority of the investigated HHD-mutations, but the different HHD variants accumulate to different densities in yeast membranes.

### Intracellular localization of HHD-hSPCA1a variants is affected by calcium homeostasis

**Figure 9:**
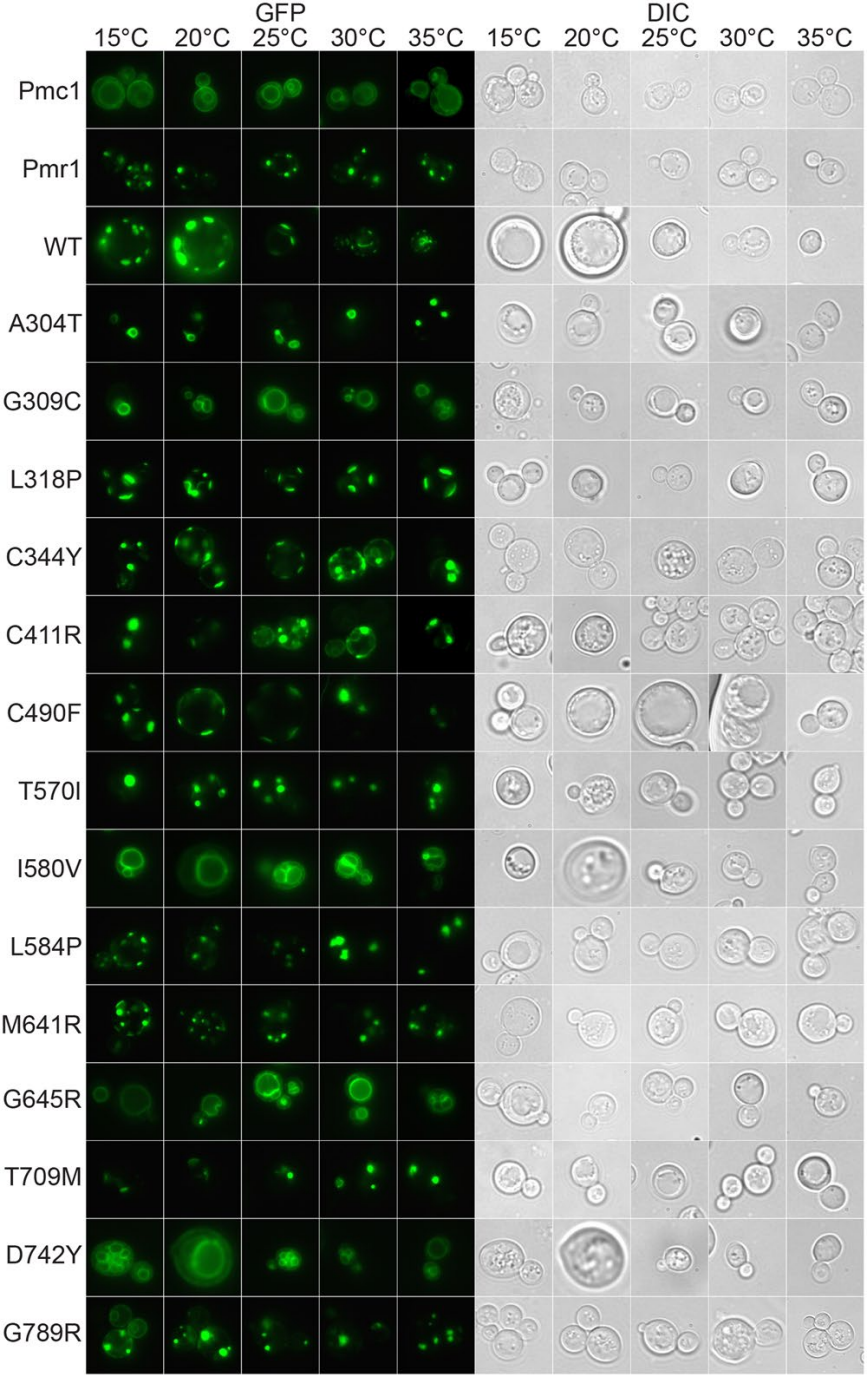
GFP tagged HHD hSPCA1a variants mislocalize in the *pmc1*Δ *pmr1*Δ *cnb1*Δ yeast strain PAP4920. Live cell bioimaging of yeast strain PAP4920 expressing C-terminally GFP tagged hSPCA1a variants, the endogenous Pmr1 or Pmc1 from the replicative vector pPAP4997 at 15 °C, 20 °C, 25 °C, 30 °C or 35 °C. Yeast cultures were inoculated in standard minimal medium supplemented with 20 mM CaCl_2_ and galactose as sole carbon source in shake flasks to OD_450_ = 0.05 at 15 °C, 20 °C, 25 °C, 30 °C and 35 °C. Live cell bio imaging was performed once OD_450_ = 1–1.5. All images were taken at 1000x magnification. Left part of the figure (GFP) shows GFP fluorescence while the right side (DIC) shows the same cells imaged with differential interference contrast. The single images shown represent one out of at least five.

**Figure 10:**
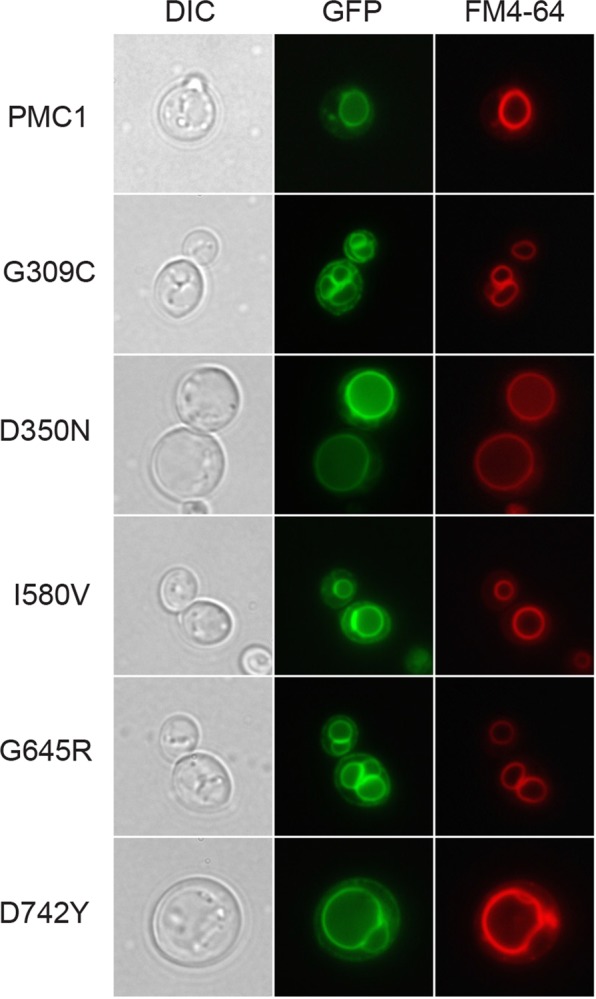
HHD variants G309C, I580V, G645R, D742Y and the enzymatically inactive D350N localize to the vacuolar membrane in the *pmc1*Δ *pmr1*Δ *cnb1*Δ strain PAP4920. Live cell bioimaging of strain PAP4920 expressing the indicated C-terminally GFP tagged hSPCA1a variants or the endogenous Pmc1 at 30 °C. Cells were grown as described in Fig. 9 and stained with the vacuolar-specific dye FM4-64 as described in Materials and Methods prior to bioimaging. All images were taken at 1000x magnification. Left column shows differential interference contrast (DIC). The middle column (GFP) shows GFP fluorescence in the same cells. The third column (FM4-64) shows the same cells dyed with FM4-64. The single images shown represent one out of at least five.

It may be anticipated that localization of hSPCA1a to the same intracellular compartment as the endogenous Pmr1 protein is a requirement for functional complementation of the calcium and manganese related phenotypes exposed by the *pmc1*Δ *pmr1*Δ *cnb1*Δ strain. To determine sub-cellular localization of wt and HHD hSPCA1a variants we expressed GFP tagged hSPCA1a variants and the endogenous Pmr1 and Pmc1 at 15 °C, 20 °C, 25 °C, 30 °C and 35 °C in the *pmc1*Δ *pmr1*Δ *cnb1*Δ strain. Live cell bioimaging of yeast cells producing GFP tagged Pmr1 or Pmc1 (Fig. 9) revealed localization to small punctuate structures scattered in the cytosol and the vacuolar membrane, respectively. The former is a characteristic appearance for yeast Golgi^17,31^. The live cell bioimaging data in Fig. 9 show that all HHD-hSPCA1a-GFP fusions except G309C, I580V, G645R and D742Y localized similarly to wt hSPCA1a-GFP and Pmr1-GFP. G309C, I580V, G645R and D742Y primarily revealed a Pmc1-like distribution, i.e. typical vacuolar membrane localization. To uniquely identify the vacuolar membrane we stained yeast cells expressing each of these variants with the specific vacuolar membrane stain FM4-64 (Fig. 10). Activity of the hSPCA1a protein does not seem to cause mislocalization as I580V shows wt-like activity, G309C only have minor Ca^2+^-transport activity and no Mn^2+^-transport activity and G645R and D742Y are devoid of activity. To be sure to have a completely inactive mutant we included hSPCA1a variant D350N that is completely devoid of any transport capacity^44,64,65^. As can be seen from Fig. 10, D350N accumulated in the vacuolar membrane as did the endogenous Pmc1.

**Figure 11:**
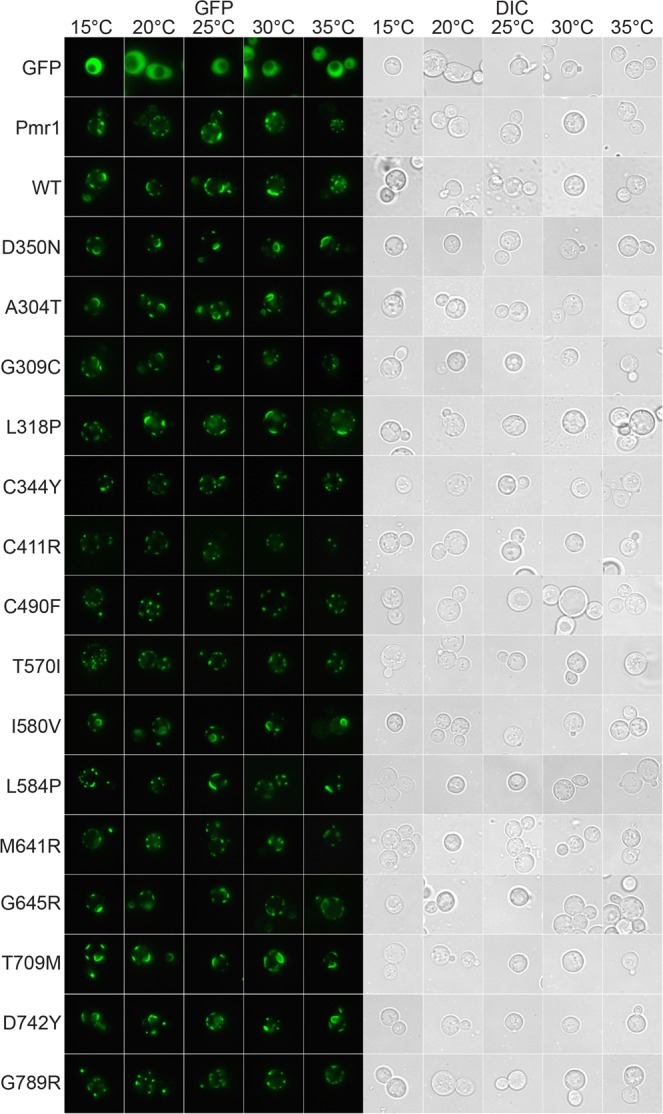
GFP tagged HHD hSPCA1a variants localizes correctly to the secretory pathway in wt yeast strain PAP1500. Live cell bioimaging of yeast strain PAP1500 expressing C-terminally tagged hSPCA1a variants, the endogenous Pmr1 or Pmc1 from the replicative vector pPAP4997 at 15 °C, 20 °C, 25 °C, 30 °C or 35 °C. Yeast cultures were grown as described in Fig. 9. All images were taken at 1000x magnification. Left part of the figure shows GFP fluorescence while the right side shows the same cells visualized with differential interference contrast (DIC). The single images shown represent one out of at least five.

To clarify whether mislocalization of the four HHD variants in some way reflects altered calcium homeostasis and/or manganese homeostasis we expressed wt hSPCA1a and all HHD variants in the wt yeast strain PAP1500^65^. This yeast strain carries intact *PMR1*, *PMC1* and *CNB1* genes and therefore is anticipated to have a normal Ca^2+^ and Mn^2+^ homeostasis, independent of the performance of the expressed hSPCA1a (Fig. 11). As can be seen from Fig. 11, all GFP tagged hSPCA1a variants localized correctly to the Golgi compartment, as did the GFP tagged Pmr1. As expected, the GFP tagged Pmc1 localized to the vacuolar membrane. This indicates that an altered Ca^2+^ and/or Mn^2+^ homeostasis may cause the observed vacuolar mislocalization of some of the HHD-hSPCA1a variants.

**Figure 12:**
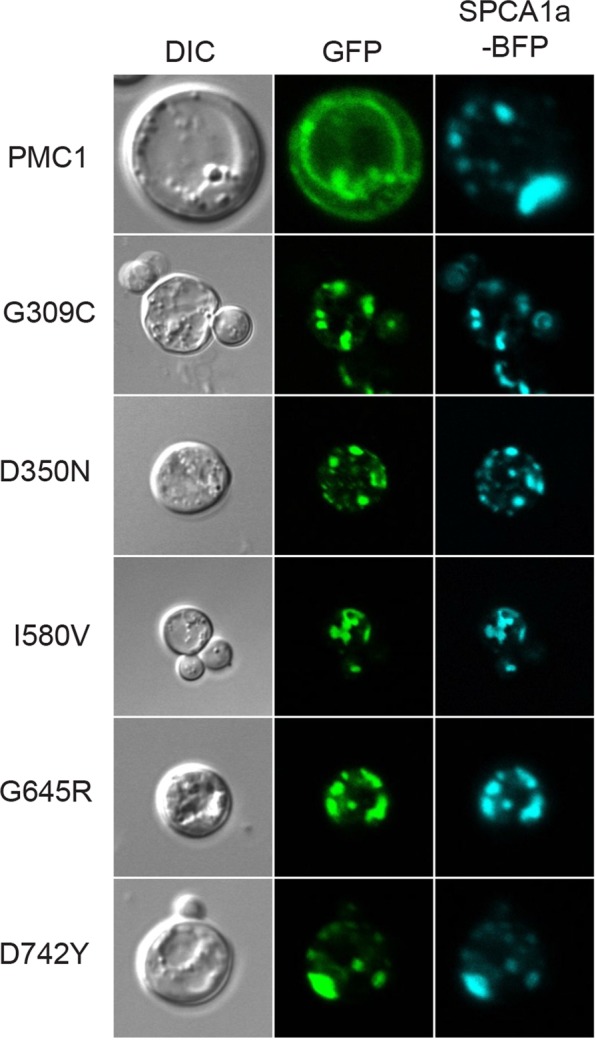
Localization of G309C, I580V, G645R, D742Y and the enzymatically inactive D350N in the *pmc1*Δ *pmr1*Δ *cnb1*Δ strain PAP4920 is rescued by co-expression of wt hSPCA1a. Live cell bioimaging of yeast strain PAP4920 co-expressing BFP tagged wt hSPCA1a and GFP tagged hSPCA1a variants or the endogenous Pmc1 from the replicative vector pPAP4997 at 30 °C. Cells were grown as described in Fig. 9. All images were taken at 1000x magnification. Left column shows differential interference contrast (DIC). The middle column (GFP) shows GFP fluorescence in the same cells. The third column (SPCA1a-BFP) shows BFP fluorescence in the same cells. The single images shown represent one out of at least five.

**Figure 13:**
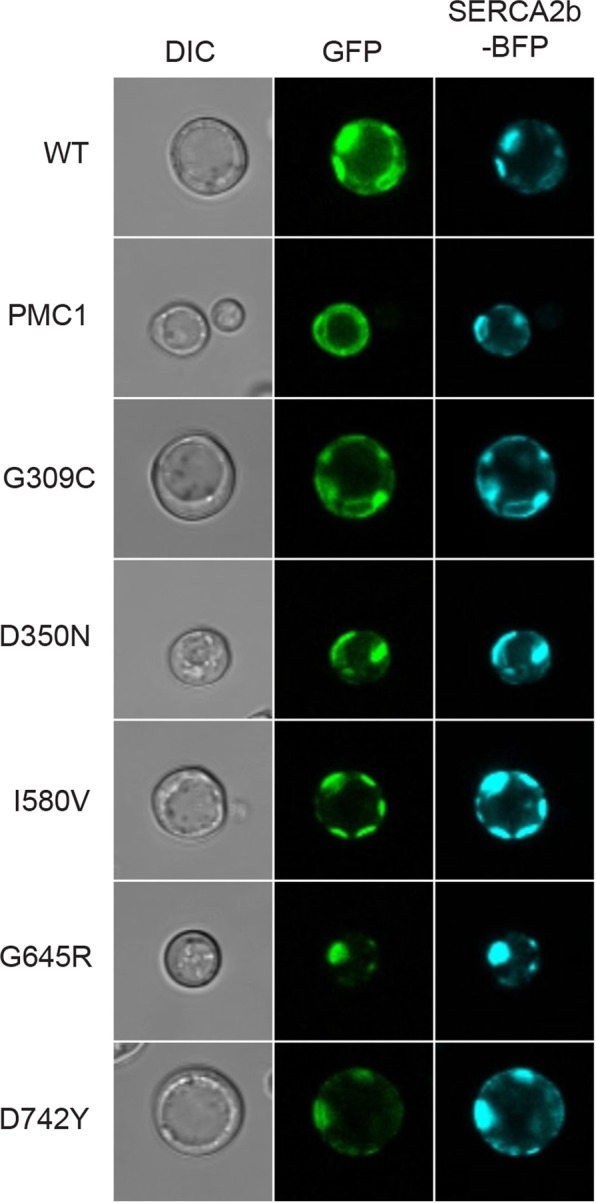
Localization of G309C, I580V, G645R, D742Y and the enzymatically inactive D350N in the *pmc1*Δ *pmr1*Δ *cnb1*Δ strain PAP4920 is rescued by co-expression of wt hSERCA2b. Live cell bioimaging of yeast strain PAP4920 expressing BFP tagged SERCA2b and GFP tagged hSPCA1a variants or the endogenous Pmc1 from the replicative vector pPAP4997 at 30 °C. Cells were grown as described in Fig. 9. All images were taken at 1000x magnification. Left column shows differential interference contrast (DIC). The middle column (GFP) shows GFP fluorescence in the same cells. The third column (SERCA2b-BFP) shows BFP fluorescence in the same cells. The single images shown represent one out of at least five.

To further clarify whether mislocalization of HHD variants G309C, I580V, G645R and D742Y as well as the functionally inactive D350N reflects altered Ca^2+^ homeostasis and/or Mn^2+^ homeostasis we co-expressed BFP tagged wt hSPCA1a and GFP tagged versions of the five mislocalized hSPCA1a variants from Fig. 10 in the triple *pmr1*Δ *pmc1*Δ *cnb1*Δ strain. Figure 12 shows that co-expression of wt hSPCA1a-BFP prevented the GFP tagged variants from accumulating in the vacuole and instead caused localization in the same Golgi compartment as wt hSPCA1a-BFP. To rule out that restoration of correct localization was due to oligomerization of wt- and HHD-hSPCA1a proteins and to clarify whether mislocalization was due to altered Ca^2+^ or Mn^2+^ homeostasis, we also co-expressed N-terminally BFP tagged human SERCA2b and the five mislocalized hSPCA1a variants. Data in Fig. 13 show that SERCA2b rescues localization of the mislocalized hSPCA1a proteins as it co-localizes with the four GFP tagged HHD-hSCPA1 variants and D350N, indicating that the four mislocalized HHD-hSPCA1a variants do indeed localize to the correct subcellular compartment when Ca^2+^ homeostasis is unperturbed, as SERCA2b cannot transport Mn^2+^.

### Amino acid substitutions in hSPCA1 induce the unfolded protein response

**Figure 14:**
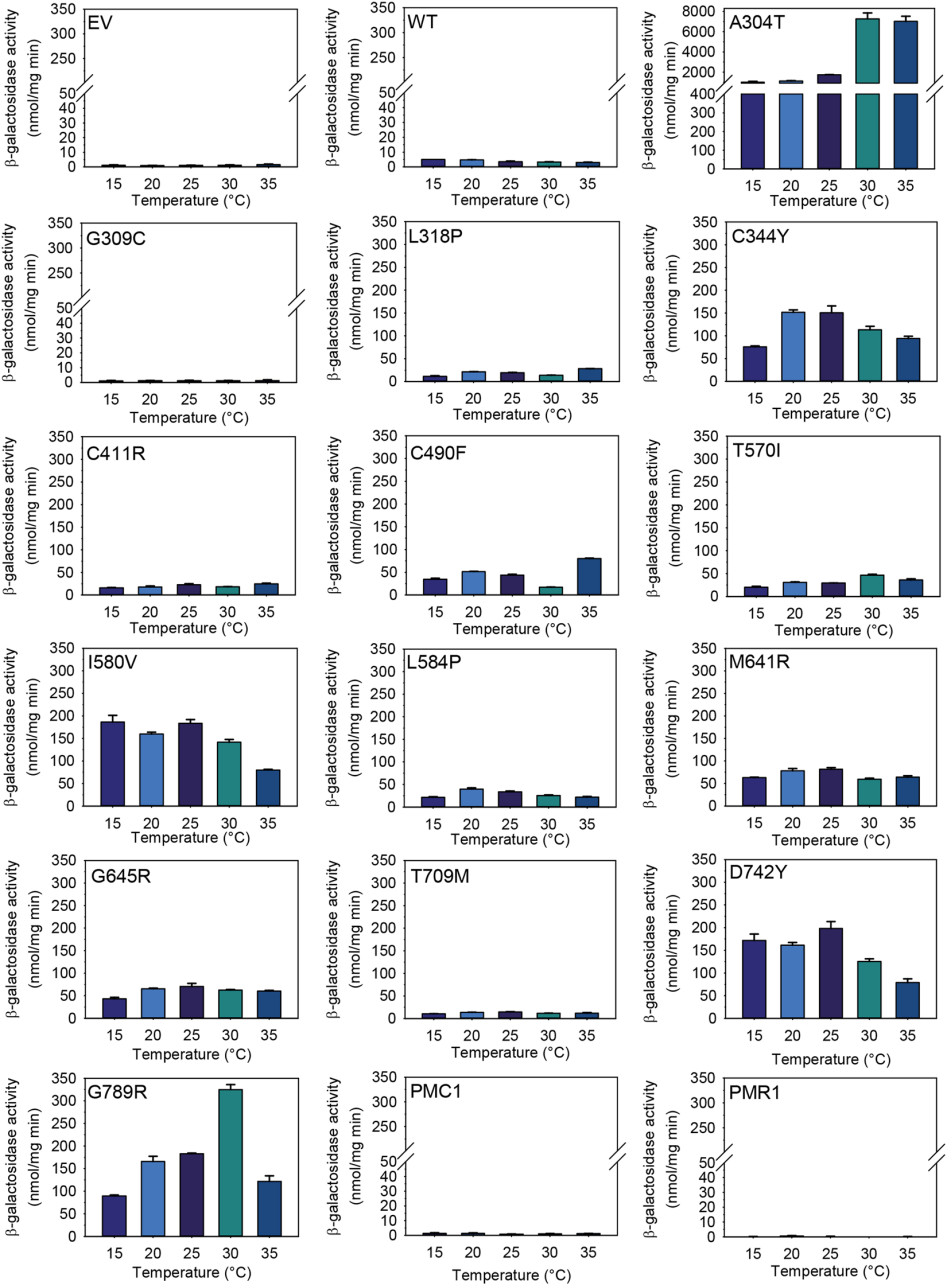
Expression of HHD-hSPCA1a-GFP in yeast strain PAP4920 induces the unfolded protein response (UPR). Yeast strain PAP4920 carries the chromosomal unfolded protein response reporter UPR-*lacZ* that can be utilized to monitor *in-vivo* protein folding. The cytosolic fractions from the yeast strains used for Fig. 8 (PAP4920 expressing Pmr1-GFP, Pmc1-GFP, wt hSPCA1a-GFP or HHD-hSPCA1a-GFP from the replicative vector pPAP4997) were analyzed for β-galactosidase activity as described in Materials and Methods. Yeast cultures were inoculated in standard minimal medium supplemented with 20 mM CaCl_2_ and galactose as sole carbon source in shake flasks at 15 °C, 20 °C, 25 °C, 30 °C and 35 °C at OD_450_ = 0.05. Cells were harvested when OD_450_ = 1–1.5. Results represent mean and standard deviations from three estimates.

As a tool to monitor whether *in vivo* folding of the fourteen clinically identified HHD hSPCA1a variants was compromised relative to that of the wt, we expressed the previously described GFP tagged versions of wt hSPCA1a, the fourteen HHD variants and the endogenous Pmr1 and Pmc1 proteins at 15 °C, 20 °C, 25 °C, 30 °C and 35 °C in the *pmc1*Δ *pmr1*Δ *cnb1*Δ yeast strain PAP4920 that carries an unfolded protein response reporter, *UPR-lacZ* in its chromosome. Expression was performed in presence of 20 mM CaCl_2_ and 0.24 µM MnSO_4_^59^ to make yeast growth independent of the Ca^2+^-Mn^2+^ATPase activity of each expressed HHD-variant and to supply MnSO_4_ at a non-limiting and non-toxic concentration. Furthermore, inclusion of high concentration of Ca^2+^ to the growth medium reduces the chronically high UPR of *pmr1*Δ strains to wt-level^61^. Data in Fig. 14 show that expression of GFP tagged Pmr1, Pmc1, wt hSPCA1a or no hSPCA1a induced a very low and temperature independent unfolded protein response. It can be seen from Fig. 14 that apart from variant G309C all HHD variants evoked an unfolded protein response higher than wt, demonstrating that these HHD variants to various degrees are recognized by the protein folding quality control in the ER. Based on the level of induced UPR we divided the variants into low- and high-UPR inducers, respectively (Table 1). Members of the first category include variants G309C and T709M, along with the empty vector, Pmc1 and Pmr1 which all induced UPR to a level below that of the wt or up to 2 times that of wt. The remaining 12 variants all induced UPR to a level at least twice that of the wt. Most of the HHD variants investigated in the present study did not show a pronounced temperature-dependent effect on UPR induction.

To investigate the effect of having a healthy Ca^2+^-ATPase, and thus to more closely mimic the situation in HHD-keratinocytes, we co-expressed the investigated HHD-variants with either wt *ATP2C1a* or wt *ATP2A2b* in the presence of 20 mM CaCl_2_ in the growth medium. The results in Supplementary Figs S12 and S13 show that co-expression of a wt hSPCA1a or wt hSERCA2b, together with HHD-hSPCA1 prevents UPR induction. Lack of UPR in the presence of a wt Ca^2+^-ATPase indicates that Ca^2+^ dyshomeostasis may contribute significantly to UPR induction in yeast expressing HHD-*ATP2C1a*.

## Discussion

HHD is caused by *ATP2C1* mutations that prevent accumulation of sufficient functional hSPCA1 in the secretory pathway. In addition to inactivation of ATPase activity an *ATP2C1* missense mutation may compromise pre-mRNA splicing, mRNA stability, protein folding, protein stability or protein localization. The focus of the present paper was to analyze how 14 *ATP2C1* missense mutations identified in HHD-patients affected Ca^2+^/Mn^2+^-ATPase activity using a yeast complementation assay. We furthermore analyzed how temperature influenced ATPase activity, protein accumulation, cellular localization and ER stress. The observed and diverse phenotypes of each mutation are summarized in Table 1.

Some of the mutants characterized in the present study have previously been investigated after expression in mammalian cells^11,66^. Table 2 shows a comparison between our results and previous results obtained from mammalian cells. Despite that different hosts were used for hSPCA1 production and different assays were applied for characterization, it can be seen that almost identical results were obtained in yeast and mammalian cells. Protein accumulation relative to wt is the same for all compared HHD variants and I580V shows wt activity in both expression systems while G309C affects Mn^2+^ interactions more than Ca^2+^. These data are in strong support for yeast being a relevant model system for studying certain aspects of HHD. Yeast however, lacks some of the proteins that interact with hSPCA1 in mammalian cells, e.g. the Orai1 Ca^2+^ channels^66^, and mutations affecting interaction with these proteins would not be picked up in a yeast based assay.

**Table 2:** Comparison of characterized HHD mutant phenotypes in *mammalian* hosts and **yeast**.

	Ca^2+^ binding^a^	Mn^2+^ binding^a^	Auto-phosphorylation	ATPase activity^b,c^		Localization^a,b,d^		Protein accumulation^a^	mRNA accumulation^a^
				Ca^2+^	Mn^2+^	Homozygous	Heterozygous^d^		
G309C	+/**ND**	*0/* **ND**	*0*^b,c^/**ND**	*0*/**+**	*ND*/**0**	*ND*/**Vacuole**	*Golgi*/**Golgi**	++/**++**	++/**ND**
C344Y	*ND*/**ND**	*ND*/**ND**	*ND*/**ND**	*ND*/**++**	*ND*/**+**	*ND*/**Golgi**	*Golgi*/**Golgi**	+/**+**	++/**ND**
C411R	*ND*/**ND**	*ND*/**ND**	*ND*/**ND**	*ND*/**+**	*ND*/**0**	*ND*/**Golgi**	*Golgi*/**Golgi**	+/**+**	++/**ND**
T570I	*ND*/**ND**	*ND*/**ND**	*ND*/**ND**	*ND*/**+**	*ND*/**0**	*ND*/**Golgi**	*Golgi*/**Golgi**	+/**+**	++/**ND**
I580V	++/**ND**	++/**ND**	++^b,c^/**ND**	++/**++**	*ND*/**++**	*ND*/**Vacuole**	*Golgi*/**Golgi**	++/**++**	++/**ND**
D742Y	*0*/**ND**	*0*/**ND**	*0*^b^/**ND**	*ND*/**+**	*ND*/**0**	*ND*/**Vacuole**	*Golgi*/**Golgi**	++/**++**	++/**ND**
G789R	*ND*/**ND**	*ND*/**ND**	*ND*/**ND**	*ND*/**0**	*ND*/**0**	*ND*/**Golgi**	*Golgi*/**Golgi**	+/**+**	++/**ND**

Values: ND, Not determined; 0, background or marginally above background; +, intermediary; ++, wt level. ^a^Data for mammalian host (COS-1 cells) obtained from Fairclough *et al*.^11^ (SPCA1d). ^b^Data for mammalian host (HEK293T cells) obtained from Smaardijk *et al*.^66^. ^c^For comparison with transiently transfected COS-1 cells^11^ or HEK293T cells^66^ only yeast data for high copy number in liquid media were included. ^d^Mammalian hosts carry at least one functional *ATP2C1* and *ATP2A2* allele (as these are not knocked out in the experiments by Fairclough *et al*. and Smaardijk *et al*.^11,66^), and are as such heterozygous when transfected with vectors carrying wt or HHD *ATP2C1*. For yeast (our assays) “heterozygous” denotes both wt *ATP2C1a*/HHD mutant co-expression and *ATP2A2b*/HHD mutant co-expression as no difference in localization of the GFP tagged HHD-SPCA1a was observed between the two experiments. All data are given as *mammalian cells*/**yeast.**

Lack of *in vivo* Ca^2+^/Mn^2+^ ATPase activity in HHD could potentially result from reduced hSPCA1 protein accumulation. Since all the investigated hSPCA1a variants, as well as the endogenous Pmr1 and Pmc1, were expressed from identical plasmids, we can assume that the rate of protein synthesis at a given temperature is the same for all the investigated proteins even though we cannot exclude that a missense mutation may affect e.g. mRNA stability. Therefore, differences in protein accumulation most probably originate from different rates of protein degradation and reflect protein stability. It is evident from Fig. 8 that at all temperatures seven of the investigated HHD-variants (C344Y, C411R, C490F, T570I, L584P, M641R and G789R) accumulated protein to a significantly lower membrane density than wt. The remaining seven variants (A304T, G309C, L318P, I580V, G645R, T709M and D742Y) accumulated protein to a level comparable to wt. However, none of these displayed a temperature dependent protein accumulation profile similar to that of the wt. Even though all hSPCA1 variants accumulated in a temperature independent way, the majority exposed a cold sensitive phenotype, showing that the accumulated protein is either non-functional or have very little ATPase activity at low temperatures. However, while HHD-mutants C344Y, C490F and L584P all accumulated protein to a lower density than wt, they were still able to complement the BAPTA-sensitive growth phenotype in liquid media almost as well as wt, indicating that the amount of functional hSPCA1a protein required for complementation is low. The other low accumulating variants all showed no or very little activity. Consequently, a reduced amount of accumulated hSPCA1a protein may not always be the cause of HHD.

Pmr1, the yeast orthologue of hSPCA1a, is located in the yeast Golgi and ER membrane^17,31–33^. We therefore anticipated that complementation requires that wt hSPCA1a and the investigated variants localize to these compartments. The bioimaging data in Fig. 9 show that wt hSPCA1a and all variants except G309C, I580V, G645R and D742Y, appear to localize similarly to Pmr1 in our *pmc1*Δ *pmr1*Δ *cnb1*Δ yeast strain. G309C, I580V, G645R and D742Y on the other hand all localized exclusively to the vacuolar membrane indicating that mis-targeting may be of importance for HHD. The fact these HHD variants exclusively accumulate in the vacuolar membrane and protein accumulation for these variants is on par with wt (Fig. 8), indicate that the proteins are not targeted to the vacuole for degradation. An interesting conclusion from these experiments is that the functional state does not dictate localization, as I580V in all assays showed wt-like complementation. Although the other mislocalization mutants all showed very little activity in our complementation assays, many other HHD variants localized correctly even though they possessed little or no complementation capacity (Table 1). The reason that I580V actually does complement the BAPTA and Mn^2+^ sensitive growth phenotypes, despite mislocalization, fits with the fact that over-expression of the vacuolar *PMC1* can compensate for loss of *PMR1* when grown in Ca^2+^-depleted media^33^, possibly because the protein is active *en route* to the vacuole, and perhaps in combination with an increased accumulation in ER and Golgi^62,67^. In this sense, the I580V variant may mimic the function of *PMC1*, but with the added functionality of Mn^2+^-transport.

Calcium homeostasis must play a role in localization as wt hSPCA1a and all mutants localized correctly in a wt yeast strain (Fig. 11) or in the calcium ATPase deficient yeast strain co-expressing either wt hSPCA1a (Fig. 12) or wt SERCA2b (Fig. 13). Since mislocalization is corrected by co-expression of SERCA2b, it must be the calcium homeostasis that is important as SERCA2b does not transport Mn^2+^.

The mislocalization we observed seems at first glance to contradict previous data^11,66^ showing wt localization of I580V and G309C in COS-1 cells and HEK293T cells, respectively. However, it should be kept in mind that these mammalian cells express endogenous wt SPCA1 as well as wt SERCA2. In our yeast model system, localization of I580V or G309C after co-expression of either wt hSPCA1a or hSERCA2b (Table 2) was identical to the localization observed in mammalian cells.

In the triple *pmc1*Δ *pmr1*Δ *cnb1*Δ yeast model system, homozygous expression of our panel of mutations seems to affect Mn^2+^ transport much more severely than Ca^2+^ transport (Table 1). As this was also observed in the single *pmr1*Δ strain, the difference is most likely attributable to the Ca^2+^ and Mn^2+^ transport capability of the HHD-hSPCA1 variants and not caused by hyper-accumulation of Ca^2+^ in the cytosol of the *pmc1*Δ *pmr1*Δ *cnb1*Δ strain. This suggests that a disturbed Mn^2+^ homeostasis may contribute significantly to HHD. In fact two of the mutations, L318P and C344Y, selectively affected Mn^2+^ transport without affecting Ca^2+^-transport. Another observation is that while most of the investigated mutations displayed a poor ability to complement the BAPTA sensitive growth deficiency of the host strain when expressed from a single copy of *ATP2C1* cDNA, most of the mutations showed significant Ca^2+^-ATPase activity when expressed from approximately twenty copies of *ATP2C1* cDNA (Figs 2 and 3). In contrast to this, Mn^2+^ toxicity could not be rescued by a twenty times increase in plasmid copy number suggesting that disturbed Mn^2+^ homeostasis may contribute significantly to HHD. Based on our results it may be interesting to measure the intracellular Mn^2+^ concentration in keratinocytes from HHD patients for comparison with that of healthy persons. The observed copy number effect on Ca^2+^-ATPase activity is in line with the general idea that haploinsufficiency is involved in HHD, while the copy number independent Mn^2+^ toxicity indicates a more complex genotype-phenotype relationship.

The results obtained after co-expression of each of the investigated HHD-alleles with wt *ATP2C1*a (hSPCA1a) (Supplementary Figs S4–S7) show that none of the investigated HHD-mutations exerted a dominant-negative effect on the Ca^2+^ or Mn^2+^ transport activity of wt hSPCA1a.

Five of the investigated amino acid substitutions are located in the transmembrane part of hSPCA1 and four of these (A304T, G309C, T709M and D742Y) are located in or close to the predicted ion-binding site created by amino acids in TM4, 5 and 6. G789R is located in TM7. None of the five mutations conferred Mn^2+^-tolerance irrespective of gene copy number, but all five conferred BAPTA sensitivity in a copy number dependent way. Each of the five mutations introduces a more bulky side chain and A304T, G309C and G789R additionally contributes a more polar side chain. The selective effect on Mn^2+^-ATPase activity may be due to altered geometry in the cation site that directly affects binding or release of Mn^2+^ more than of Ca^2+^, potentially due to the fact that the ionic radius of Mn^2+^ is considerably smaller than that of Ca^2+^ (0.8 Å vs. 0.99 Å respectively^68^) and therefore interactions with the binding site may affect Mn^2+^ more that Ca^2+^. It may seem counterintuitive that mutations preclude transport of the smaller Mn^2+^ ion, but not the larger Ca^2+^ ion. However, ion selectivity reflects the balance between the cost of ion dehydration and the benefits of interaction with the ion binding site^69^. As exemplified in K^+^ channels this results in a 10,000 times greater affinity for the larger K^+^ ion over the smaller Na^+^ ion. It has furthermore been shown that the selectivity of the K^+^ channel can be manipulated by adjusting the size of the ion-binding cavity^69^.

The reaction cycle of P-type ATPases involves large rearrangements and transmission of conformational changes initiated in the cytosolic A-, N- and P-domains and transduced to the membrane bound M-domain and vice-versa through long-range interactions between residues that are distantly remote in the primary structure^70–72^. Apart from amino acid substitutions in the ATP or Mg^2+^ binding sites, substitutions in one part of the protein may thus affect ATPase activity by reducing or preventing structural transmission from the cytoplasmic domains to the ion binding site in the membrane. It is well established that shortcutting the essential communication between A, N, P and M domains by limited proteolysis prevents ATPase activity^73^. Because at least some Ca^2+^-ATPase activity is preserved in several of the analyzed HHD variants, communication between the cytoplasmic part and the cation sites cannot be completely blocked; it is more likely that communication is impaired in such a way that the geometry of the cation binding site in the trans-membrane domain is altered and affects the smaller Mn^2+^ ion more than the larger Ca^2+^ -ion.

The simplest way to inactivate a protein is to interfere with its folding^74^ either by compromising the folding pathway or the stability of the finally folded structure. The circumstance that yeast can grow at a wide temperature range offers a unique opportunity to demonstrate for the first time, that nine out of the fourteen HHD mutations analyzed in the present study exposed a cold sensitive phenotype as complementation was absent at the lower temperatures (Fig. 7). Cold sensitive mutations are rare^75^ and usually result from trapping of a protein in a folding intermediate, that to various degrees prevents it from achieving the biologically active, three dimensional structure. Folding of the great majority of the HHD variants at low temperatures may thus be compromised by single amino acid substitutions. It is not obvious how cold sensitivity and the observation that wt hSPCA1a accumulates better at low temperature correlates with HHD. Even though the average skin temperature is 5–7 °C lower than the core temperature at normal ambient “comfort temperature”^76,77^ the disease exposes itself in intertriginous areas and parts of the skin exposed to friction where humidity and temperature are increased. HHD is almost exclusively pronounced in skin and extracutaneous symptoms have only been described in liver^78^. Given the importance of Ca^2+^ in a plethora of cellular processes it is surprising, that skin is the mainly affected organ in HHD. A putative explanation has been that other tissues must have compensatory Ca^2+^ transport mechanisms or that *ATP2C1* expression is upregulated in keratinocytes as a consequence of the lower Ca^2+^ content of the upper layers in the epidermis^37^.

Despite their diverse phenotypes it is very interesting that homozygous expression of twelve out of the fourteen investigated HHD variants induced an unfolded protein response, because this may pave the way for a completely new way to target HHD. ER-stress has earlier been suggested to play an important role in the development of HHD due to the important role of Ca^2+^ in the secretory pathway of eukaryotic cells^38^. ER-stress and UPR can be induced by either depleting the ER of Ca^2+^ or by bona fide protein misfolding^53,57,79–81^. Consequently, HHD mutations may induce UPR in three ways: 1) By resulting in a non-functional or poorly functioning Ca^2+^-ATPase that is unable to counteract the constant Ca^2+^-leakage out of ER; 2) By resulting in an inactive, leaky protein that allows Ca^2+^ ions to more or less passively leak out of the ER; or 3) By misfolding.

Mutations resulting in poorly functioning or non-functioning hSPCA1a are expected to behave in a similar manner to the empty vector (Fig. 14) and the enzymatically dead D350N (Supplementary Fig. S14), and should thus induce a very low level of UPR in our yeast model system. To prevent the chronically induced UPR of *pmr1*Δ yeast in low Ca^2+^ media, we supplement the growth medium with 20 mM CaCl_2_ as this fully restores growth and UPR to wt levels^60,61^. The normal feedback inhibition of HACS is also absent in our yeast strain. Addition of 20 mM CaCl_2_ to the growth medium thus creates a large enough Ca^2+^ gradient from the outside, through the cytoplasm and across the ER membrane to allow a sufficient amount of Ca^2+^ to enter the ER lumen, to allow growth and suppress UPR. It has previously been shown that mutations in *ATP2A2* can result in leaky SERCA2 proteins^82^. Under normal physiological conditions Ca^2+^ would leak out of the ER since the Ca^2+^ concentration in the ER is generally much greater than that of the cytosol. Under our growth conditions, the electrochemical gradient supports Ca^2+^ influx into the ER, and Ca^2+^ leakage out of the ER thus seems unlikely. Misfolding itself could theoretically trigger UPR by bona-fide misfolding (through the phylogenetically conserved Ire1 and BiP^83–85^) or by sequestering BiP and thereby increasing Ca^2+^ leakage through translocons^80,86,87^. As leakage out of ER is unlikely, UPR in our experiments most likely reflect misfolding of the expressed HHD-hSPCA1a variants.

Amino acids 723 to 729 (723-MNFPNPL-729, located in the TM5-TM6 loop (luminal loop 3), expose the most probable BiP binding site (Supplementary Fig. S15 and Fig. 1). It is therefore possible that structural perturbations in hSPCA1a due to amino acid substitutions may expose this BiP binding site and induce UPR. Supplementary Fig. S16 shows that TM5, along with TM7, is predicted to have the highest number of helix-helix contacts (7) of the 10 TM helices present in hSPCA1a. Thus mutations that affect membrane organization of TM helices 2, 3, 4, 7, 8, 9 or 10 could potentially interfere with TM5 and expose the BiP binding site. This may explain why HHD causing amino acid substitutions do not cluster in any particular parts of the hSPCA1 protein.

The UPR induced by homozygous expression of wt *ATP2C1a* is low and on par with that observed for the endogenous Pmc1 or Pmr1 expressed from the same vector, demonstrating that heterologous expression of the human wt *ATP2C1a* does not cause any significant increase in UPR (Fig. 14). High UPR is induced irrespective of the ability of the specific HHD variant to complement the growth phenotype of the yeast host cell (Table 1), showing that information on Ca^2+^/Mn^2+^ ATPase activity may not be sufficient to identify the cause of HHD. The fact that our data do not demonstrate any correlation between Ca^2+^ ATPase activity and induction of UPR further supports that it is most likely protein misfolding that is the cause of UPR induction. It may seem contradictory that some HHD hSPCA1a variants induce the unfolded protein response but at the same time show complementation. However, this may reflect the folding efficiency of each variant as we do not know how many functional hSPCA1 proteins are required for complementation.

The fact that most HHD-mutations do indeed induce UPR when expressed by themselves shows the relevance of our yeast model as a background-free model organism for HHD, since this effect would not be picked up in cells that harbor endogenous Ca^2+^-ATPases. While homozygous expression of HHD-hSPCA1a points to misfolding as the most probable cause for UPR induction, the results of the heterozygous co-expression of HHD-mutant *ATP2C1a* with either wt *ATP2C1a* or wt *ATP2A2b* seems to point towards Ca^2+^-dyshomeostasis in the ER as the cause for UPR. ER Ca^2+^ dyshomeostasis and protein folding are in fact highly interconnected, since ER chaperone function and thus protein folding, is indeed highly dependent on Ca^2+^ in the ER^88^. In tissues or part of tissues, where Ca^2+^ is naturally low, ER Ca^2+^ could become so low to cause the slightly more unstable HHD-SPCA1 to misfold and induce UPR. This could then initiate a negative spiral of less Ca^2+^ resulting in more unfolded proteins and so on. A consequence of UPR in mammalian cells is attenuation of translation and thus a reduction in the amount of protein entering the ER. This could reduce the number of active Ca^2+^-ATPases further acerbating the Ca^2+^ loss in the secretory pathway. Another consequence could be interference with production of proteins located in desmosomes, which often appear defective in HHD^89^.

The physiological role of both UPR and mislocalization in HHD keratinocytes remains to be investigated, but our data suggest that both could become a factor under low Ca^2+^ conditions in the ER.

An interesting phenotype displayed by several of the investigated mutations is sensitivity to Ca^2+^ as these mutants grew better in galactose medium containing a little BAPTA than in the same media containing 20 mM CaCl_2_ or 0 mM BAPTA. The latter medium is not supplemented with Ca^2+^ and thus only contains Ca^2+^ present as contamination in the agar and other media constituents. Ca^2+^ sensitivity has previously been described for yeast *pmr1* mutants^39,40,90^ and *pmc1* mutants^40,60,91^ but at much higher Ca^2+^ concentrations (200 mM) and both single mutants grow well at 100 mM CaCl_2_^40^. *pmc1*Δ *pmr1*Δ *cnb1*Δ yeast have previously been shown to grow well at 20 mM CaCl_2_^60^ and up to 200 mM CaCl_2_^92^. The negative control (empty vector) did not display Ca^2+^ sensitivity in our experiments. Addition of 20 mM CaCl_2_ to the medium did not have an effect in the presence of glucose (i.e. when *ATP2C1* is not expressed). Interestingly, only the replicative variants displayed Ca^2+^ sensitivity on media containing galactose and 20 mM CaCl_2_, indicating that it is the combination of high expression and the specific HHD variant that is responsible for this phenotype, as neither expression of wt nor the negative control conferred any Ca^2+^ sensitivity. We did not observe a clear correlation between Ca^2+^ sensitivity and other phenotypes investigated in our study and it is thus difficult to draw any conclusions on what may cause the observed Ca^2+^ sensitivity. However, keratinocytes defective in hSPCA1 display an inability to properly accumulate Ca^2+^ upon increases in extracellular Ca^2+^^2,16^, and a similar observation has also been made with yeast defective in Pmr1^29,90^. Ca^2+^ sensitivity has even been suggested to be the most relevant phenotype for yeast *pmr1* mutants in resembling keratinocyte pathology^29^.

In conclusion, results obtained in our yeast model system demonstrate that while the phenotypes exposed by our panel of HHD mutants are very diverse, UPR induction (12 out of 14) and Mn^2+^ sensitivity (12 out of 14) are the two most dominating phenotypes. This may indicate that HHD disease is a conformational disease and Mn^2+^ homeostasis may be more affected than Ca^2+^ homeostasis. A new approach towards treatment of HHD may thus focus on development of chemical chaperones to combat the hSPCA1 folding problems we have demonstrated.

## Methods

### Yeast strains

The yeast strain K616 (*ade2*Δ*1 can1-100 his3*Δ *11*.*15 leu2*Δ *3*.*112 trp1*Δ *1 ura3*Δ *1 pmc1::TRP1 pmr1::HIS3 cnb1::LEU2*)^60^ was a kind gift from Kyle Cunningham, John Hopkins University, USA. Strain PAP4920 was generated by insertion of an unfolded protein response reporter, *UPR-lacZ*, into the *ade2*Δ*1* locus of K616. Strain PAP9588 was constructed by inserting a nourseothricin resistance gene from plasmid pAG25^86^ into the *TRP1* gene of PAP4920 by homologous recombination. The *UPR-lacZ* reporter is identical to that previously described^58^ except that the *ADE2* marker substituted for the *LYS2* marker. PAP1500 (α *ura3–52 trp1:: GAL10-GAL4 lys2–801 leu2*Δ*1 his3*Δ*200 pep4::HIS3 prb1*Δ*1*.*6* *R can1 GAL*) has been described previously^65^. Yeast strain Y04534 (BY4741; MATa*; ura3*Δ*0; leu2*Δ*0; his3*Δ*1; met15*Δ*0; YGL167c::*kanMX4) was purchased from Euroscarf.

### Plasmid constructions

The replicative Gateway® compatible expression vector pPAP4997 was generated by insertion of a *Sac*I-attR1- *ccdB*- *Cm*^*R*^ -attR2-*Hin*dIII fragment PCR amplified from pDEST15 (Invitrogen, USA) into similarly digested pEMBLyex4^93^. The integrative Gateway® compatible expression vector pPAP5480 was constructed by relegation of *Mfe*I digested pPAP4997. The replicative Gateway® compatible expression plasmids pPAP7177 and pPAP8754 were constructed by replacing the URA3 gene in pPA4997 with the ADE2 gene or the TRP1 gene, respectively.The integrative Gateway® compatible expression vector pPAP7010 was generated by inserting an *Apa*I-CYC-GAL-promoter- attR1-*ccdB*-*Cm*^*R*^-attR2-*Bgl*II PCR fragment amplified from pPAP4997 into *Apa*I, *Bam*HI digested pRS402^94^. Plasmids expressing either wt or amino acid substituted human hSPCA1a were generated by overlap extension PCR^95^ using an *ATP2C1a* cDNA clone obtained from Invitrogen, USA. Final PCR products were transferred by Gateway® Technology to pDONOR221 (Invitrogen, USA). After confirmation of the correct nucleotide sequence, all *ATP2C1* alleles were transferred to pPAP4997 and pPAP5480 by LR-recombination. wt *ATP2C1* was furthermore LR recombined into pPAP7010, pPAP7177 and pPAP8754. C-terminal *in frame* fusions to yEGFP^96^ was created by *in vivo* homologous recombination in yeast between *Sac*I or *Hin*dIII digested *ATP2C1* expression plasmids and yEGFP PCR fragments. C-terminal tagging of wt *ATP2C1* with BFP was generated by *in vivo* homologous recombination in yeast between *Sal*I, *Hin*dIII digested pPAP8754, a wt *ATP2C1* PCR fragment and a codon optimized BFP (Gateway® TagBFP-AS-C, Evrogen, Russia) PCR fragment.

### Complementation on agar plates

Yeast cells inoculated from −80 °C stock were grown at 30 °C in liquid synthetic minimal medium containing 2% glucose as sole carbon source and 20 mM CaCl_2_. Cultures maintained in the exponential growth phase were harvested, washed five times in sterile 18 mΩ water and subsequently re-suspended in 18 mΩ water to an OD_450_ = 0.5. 7 µl of this cell-suspension was spotted on minimal plates containing galactose as sole carbon source (SG plates) and either 20 mM CaCl_2_, BAPTA or MnSO_4_. Sterile filtered CaCl_2_ (from 1 M stock in dH_2_O), BAPTA (from 15 mM stock in 100 mM TES/KOH, pH = 6) or MnSO_4_ (from 100 mM stock in TES/KOH pH = 6) was added to the media to obtain the required concentrations. Plates were incubated at 15 °C, 20 °C, 25 °C, 30 °C or 35 °C, inspected daily and photographed.

### Complementation in liquid media

Yeast cells were cultured from −80 °C stock as above. Complementation assays were performed in clear 96-well plates (well capacity: 400 µl). Each well contained 200 µl yeast culture. Medium was made from 100 µl 2x SG medium and 80 µl of CaCl_2_, BAPTA or MnSO_4_ (all dissolved in 100 mM TES/KOH, pH = 6) to the desired final concentrations. 20 µl of a cell suspension with OD_450_ = 0.5 in dH_2_O was added to each well. Plates were incubated at the desired temperature and OD_450_ of the cultures measured in a plate-reader (Multiskan RC, Thermo Labsystems) at appropriate time intervals. BAPTA (1, 2-bis(o-Aminophenoxy)ethane-N,N,N’,N’-tetraacetic Acid) was purchased as tetrasodium salt from Merck (cat.no.: 196418).

### Growth of cells for quantitative fluorescence and *UPR-lacZ* measurements

Yeast cells were grown in SD medium supplemented with 20 mM CaCl_2_ and washed five times in 18 mΩ H_2_O. Each culture was used to inoculate shake flasks containing 50 ml of galactose minimal medium and either 20 mM CaCl_2_, 0 mM CaCl_2_ or 0.06 mM BAPTA to OD_450_ = 0.05. Shake flask were inoculated at 15 °C, 20 °C, 25 °C, 30 °C or 35 °C. Cultures were harvested when OD_450_ reached 1 to 1.5, washed in 18 mΩ H_2_O and immediately frozen at −80 °C. Crude yeast membranes and cytosolic fractions were isolated as previously described^65^.

### Protein and enzymatic assays

Protein concentrations in cytosolic- and membrane preparations were determined by the BCA method^97^ according to the manufacturer’s specifications (Sigma-Aldrich, USA). β-galactosidase activities were determined in purified cytosolic preparations as described previously^58^.

### Bioimaging of live yeast cells

C-terminally GFP/BFP tagged hSPCA1a proteins, N-terminally BFP tagged SERCA2b and FM4-64 stained cells^98^ were visualized at 1,000 x magnification with a Nikon Eclipse E600 fluorescence microscope equipped with an Optronics Magnafire model S99802 camera attached or a Leica SP5-X confocal microscope.

### Quantification of *ATP2C1a*-GFP protein

The density of *ATP2C1a*-GFP protein in crude yeast membranes was quantified using a standard curve made from purified GFP protein as described previously^99^.

### Bioinformatics

Prediction of BIP binding sites in ER resident parts of *ATP2C1*was performed according to Blond-Elguindi *et al*.^100^. ER resident parts were identified by the TMHMM software^101^ while potential interactions between transmembrane helixes were predicted by the TMHIT program^102^.

## Supplementary information

Characterization of Hailey-Hailey Disease-mutants in presence and absence of wild type SPCA1 using Saccharomyces cerevisiae as model organism

## Data Availability

The datasets generated during and/or analysed during the current study are available from the corresponding author on reasonable request.
