# Author Correction: Characterization of Hailey-Hailey Disease-mutants in presence and absence of wild type SPCA1 using *Saccharomyces cerevisiae* as model organism

**DOI:** 10.1038/s41598-020-66087-6

**Published:** 2020-06-04

**Authors:** Daniel Muncanovic, Mette Heberg Justesen, Sarah Spruce Preisler, Per Amstrup Pedersen

**Affiliations:** Department of Biology, August Krogh Building, University of Copenhagen, Universitetsparken 13, 2100 Copenhagen, OE Denmark

Correction to: *Scientific Reports* 10.1038/s41598-019-48866-y, published online 27 August 2019

This Article contains errors.

As a result of an error during figure assembly, in Figure 6 an image for Galactose+0.9mM BAPTA at 35°C is a duplicate of image for Galactose+1.0mM BAPTA at 35°C, and Galactose+1.2mM BAPTA at 35°C is a duplicate of image for Galactose+1.1mM BAPTA at 35°C. The correct Figure 6 is shown below as Figure 1.

**Figure 1:**
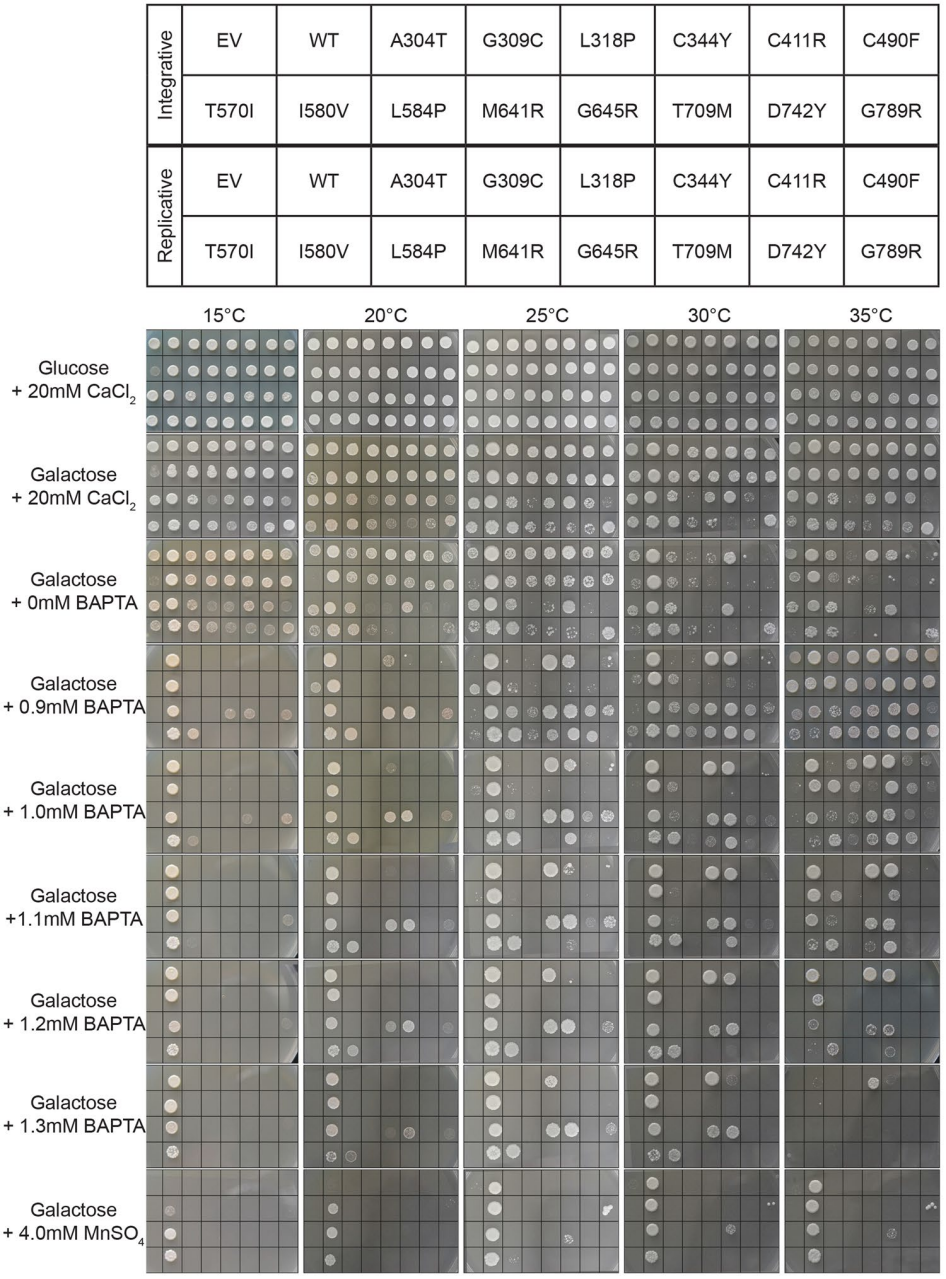
.

This error affects also temperature profiles shown in Figure 7, which is based on the data from Figure 6. The correct Figure 7 is shown below as Figure 2.

**Figure 2:**
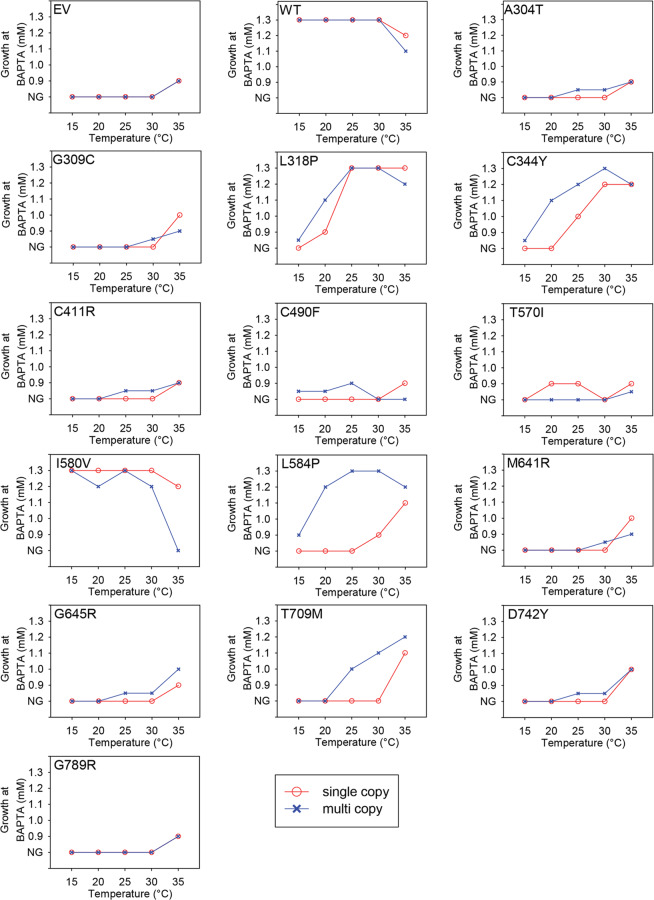
.

Finally, in the Results, subheading 'Cold sensitivity is a common phenotype exposed by HHD-mutants',

“The ability of L318P and I580V to complement is considerable reduced at 35°C compared to 30°C, while complementation by M641R is marginally reduced at 35°C.”

should read:

“The ability of L318P and I580V to complement is considerably reduced at 35°C compared to 30°C, while complementation by M641R is marginally increased at 35°C. Complementation by M641R showed a marginal reduction at 35°C in supplementary figure S10, while it shows a diminutive increase in the spot assay.”

And in the same section,

“HHD-variants A304T, C411R, M641R and G645R also displayed a CS phenotype, albeit less clear than the HHD-mutations mentioned above. Only substitutions C490F and I580V conferred a heat sensitive phenotype, the former only when expressed from the multi copy plasmid and the latter most pronounced when expressed from the multi copy plasmid. Amino acid substitutions G309C and T570V each showed a more complex phenotype. G309C only complemented weakly at 30°C and 35^°^C at a copy number of twenty and one respectively, indicating a CS-like phenotype; T570I only showed complementation at 20^°^C and 25^°^C and only after expression from the single copy plasmid, indicating a mixed temperature sensitive phenotype.”

should read:

“HHD-variants A304T, G309C, C411R, M641R and G645R also displayed a CS phenotype, albeit less clear than the HHD-mutations mentioned above. Only substitutions C490F and I580V conferred a heat sensitive phenotype, the former only when expressed from the multi copy plasmid and the latter most pronounced when expressed from the multi copy plasmid. Amino acid substitution T570I showed a more complex phenotype as it showed complementation at 20°C and 25°C only after expression from the single copy plasmid, indicating a mixed temperature sensitive phenotype.”

The overall conclusions of the Article are unaffected by these changes.

