# Supplementary material for: Characterization of Hailey-Hailey Disease-mutants in presence and absence of wild type SPCA1 using Saccharomyces cerevisiae as model organism
Source: Sci Rep. 2019 Aug 27;9:12442. doi: 10.1038/s41598-019-48866-y (PMC6712213; doi:10.1038/s41598-019-48866-y)
Supplement: Supplementary file 1 — Characterization of Hailey-Hailey Disease-mutants in presence and absence of wild type SPCA1 using Saccharomyces cerevisiae as model organism [file 41598_2019_48866_MOESM1_ESM.pdf]

Supplementary Information

(Supplementary Figures S1-S16)

Characterization of Hailey-Hailey Disease-mutants in presence and absence of wild type SPCA1 using *Saccharomyces cerevisiae* as model organism.

Daniel Muncanovic, Mette Heberg Justesen, Sarah Spruce Preisler and Per Amstrup Pedersen*

Supplementary Figure Legends

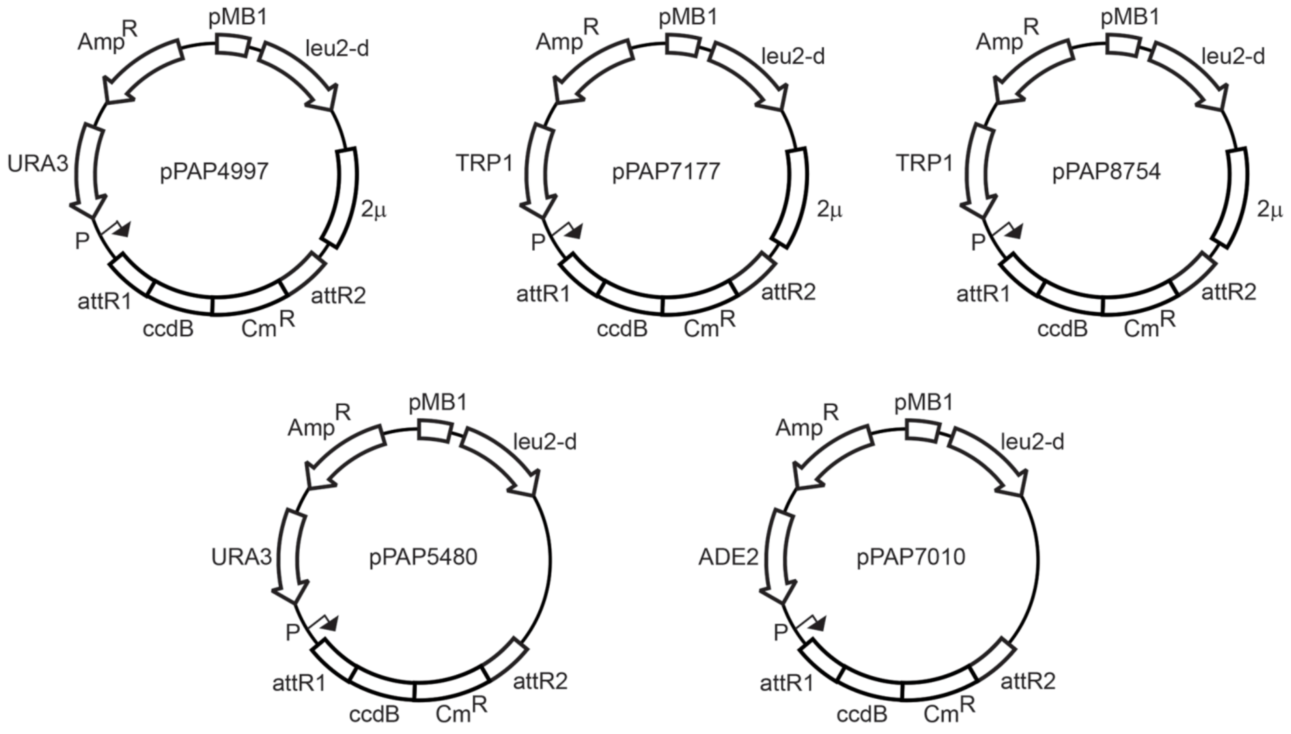

Supplementary Figure S1. Structural maps of the five plasmids used in the present work to express *ATP2C1a* (hSPCA1a) or *ATP2A2b* (hSERCA2b) alleles. pPAP4997 is a Gateway® compatible replicative expression plasmid while pPAP5480 is an integrative version of pPAP4997. pPAP7177 and pPAP8754 are identical to pPAP4997 except that ADE2 or *TRP1* has substituted for *URA3,* respectively. pPAP7010 is an integrative version of pPAP7177. pMB1, *E.coli* pMB1 origin of replication; 2μ, yeast origin of replication; f1, the phage f1 origin of replication; Amp^R, Ampicillin resistance gene; *URA3*, yeast orotidine 5-phosphate decarboxylase gene; *leu2-d*, a poorly expressed allele of the yeast β-isopropylmalate dehydrogenase gene; *TRP1*, yeast Phosphoribosyl anthranilate isomerase gene; *ADE2*, yeast Phosphoribosylaminoimidazole carboxylase gene. P, yeast CYC-GAL promoter; attR1/attR2, attachment sites for Gateway® LR recombination; *ccdB*, an

E.coli cytotoxic lethal gene encoding an inhibitor of DNA gyrase (topoisomerase II); Cm^R, Chloramphenicol resistance gene. Maps are not drawn to scale.

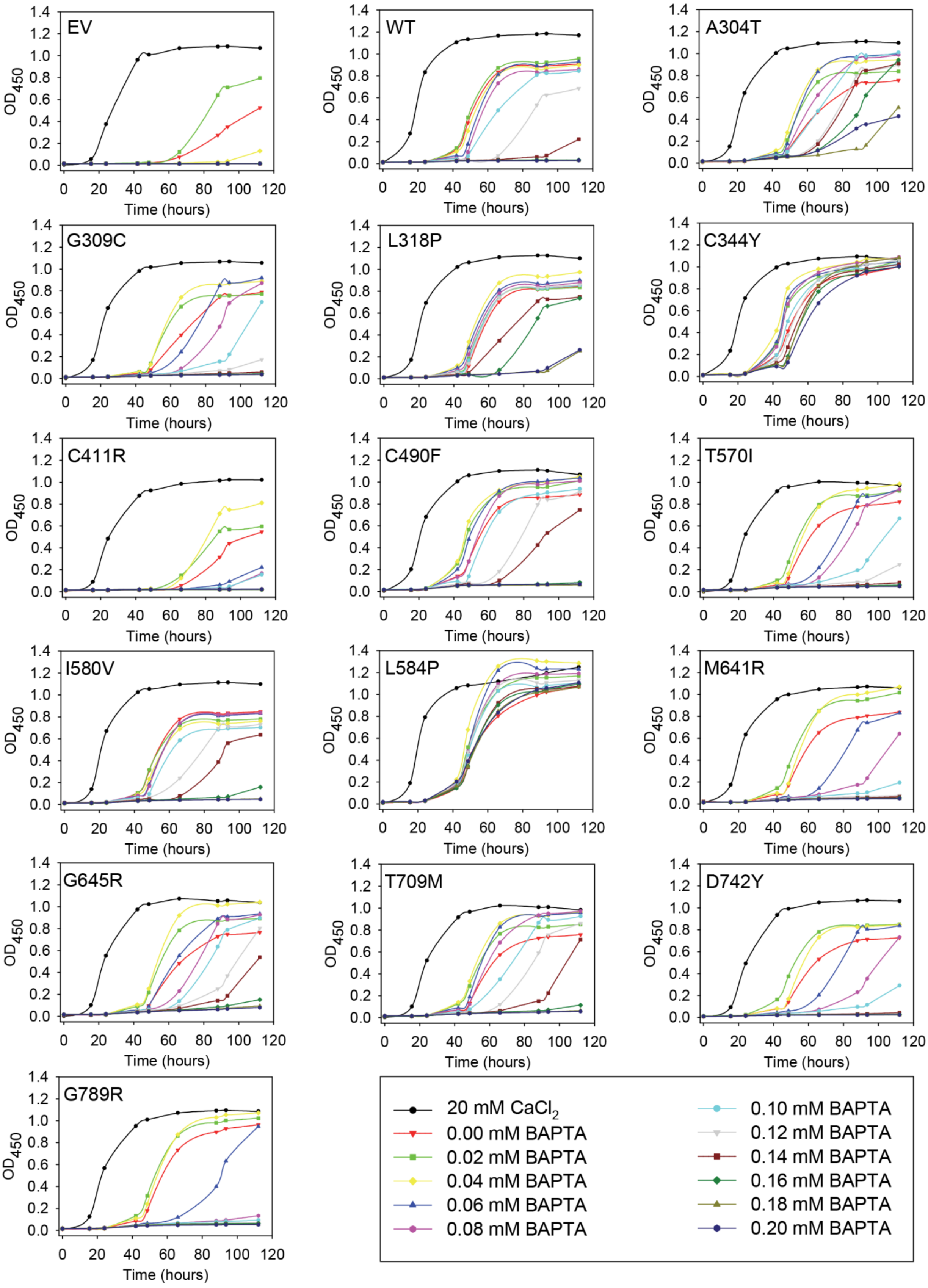
EV
WT
A304T
G309C
L318P
C344Y
C411R
C490F
T570I
I580V
L584P
M641R
G645R
T709M
D742Y
G789R
OD450
Time (hours)
0.0 0.2 0.4 0.6 0.8 1.0 1.2 1.4
0 20 40 60 80 100 120
20 mM CaCl2
0.00 mM BAPTA
0.02 mM BAPTA
0.04 mM BAPTA
0.06 mM BAPTA
0.08 mM BAPTA
0.10 mM BAPTA
0.12 mM BAPTA
0.14 mM BAPTA
0.16 mM BAPTA
0.18 mM BAPTA
0.20 mM BAPTA

Supplementary Figure S2. Complementation of the calcium dependent phenotype of the *pmr1*Δ yeast host strain by replicative hSPCA1a mutants.

Yeast strain Y04534 expressing no hSPCA1a (Empty Vector, EV), wt hSPCA1a (wt) or the indicated HHD hSPCA1a variants from the replicative vector (pPAP4997, Supplementary Fig. S1) were grown at 30°C in 96-well plates in liquid media with galactose as carbon source supplemented with 20 mM $CaCl_2$ or BAPTA concentrations ranging from 0 to 0.2 mM in increments of 0.02 mM as indicated. Each culture was grown in triplicates and the average OD_{450} at the given time was plotted with respect to time of inoculation. Growth media containing BAPTA (including 0 mM BAPTA) do not have any added Ca^{2+}.

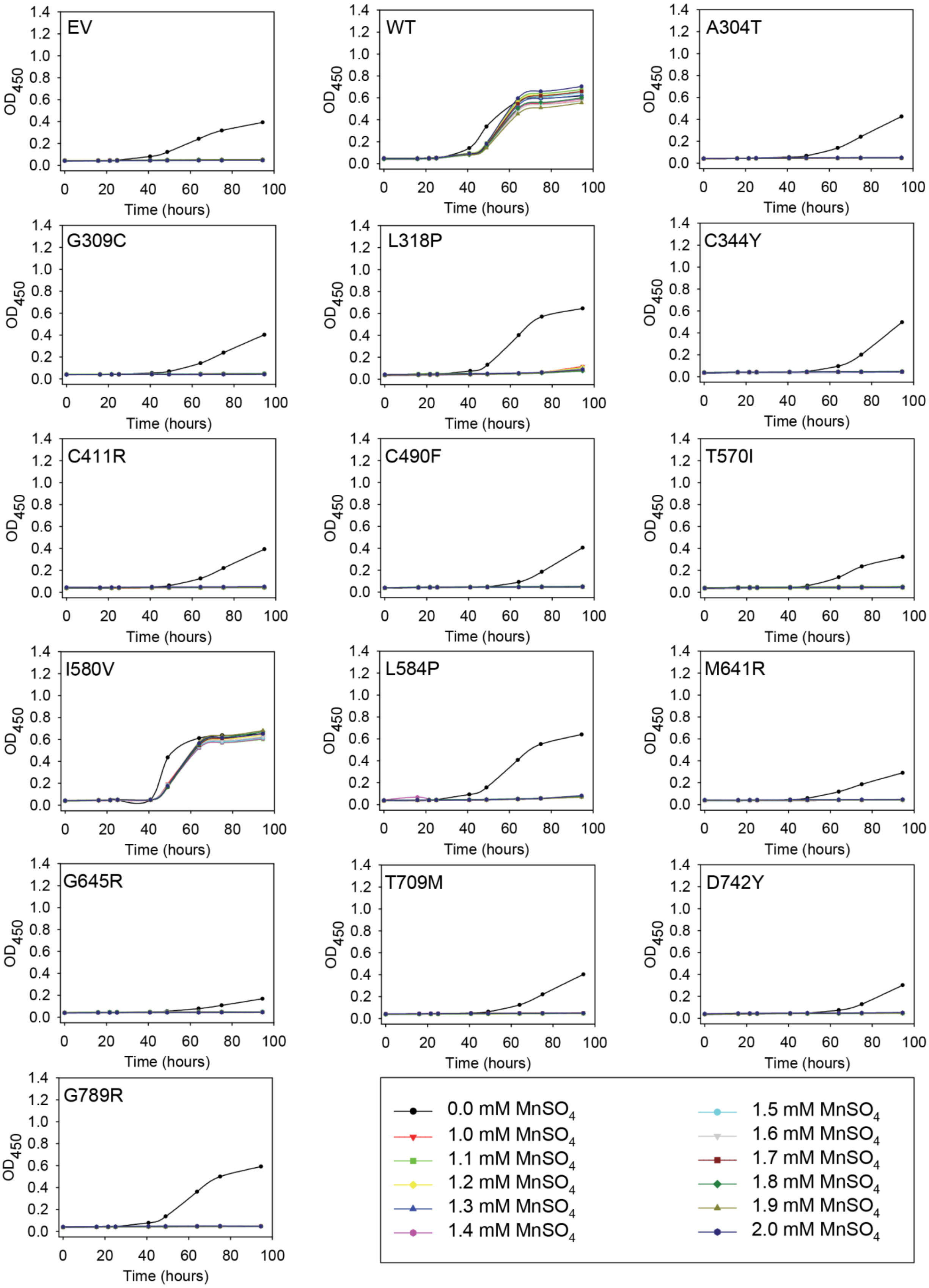
EV
WT
A304T
G309C
L318P
C344Y
C411R
C490F
T570I
I580V
L584P
M641R
G645R
T709M
D742Y
G789R
OD450
Time (hours)
0.0 mM MnSO4
1.0 mM MnSO4
1.1 mM MnSO4
1.2 mM MnSO4
1.3 mM MnSO4
1.4 mM MnSO4
1.5 mM MnSO4
1.6 mM MnSO4
1.7 mM MnSO4
1.8 mM MnSO4
1.9 mM MnSO4
2.0 mM MnSO4

Supplementary Figure S3. Complementation of the manganese sensitive phenotype of the *pmr1*Δ yeast host strain by replicative hSPCA1a mutants.

Yeast strain Y04534 expressing no hSPCA1a (Empty Vector, EV), wt hSPCA1a (WT) or the indicated HHD hSPCA1a variants from the integrative vector (pPAP5480, Supplementary Fig. S1) were grown at 30°C in 96-well plates in liquid media with galactose as carbon source supplemented with 20 mM $CaCl_2$, and $MnSO_4$ ranging from 0 to 2.0 mM in increments of 0.1 mM as indicated. Each culture was grown at 30°C, in triplicates, and average OD_{450} was plotted with respect to time of inoculation.

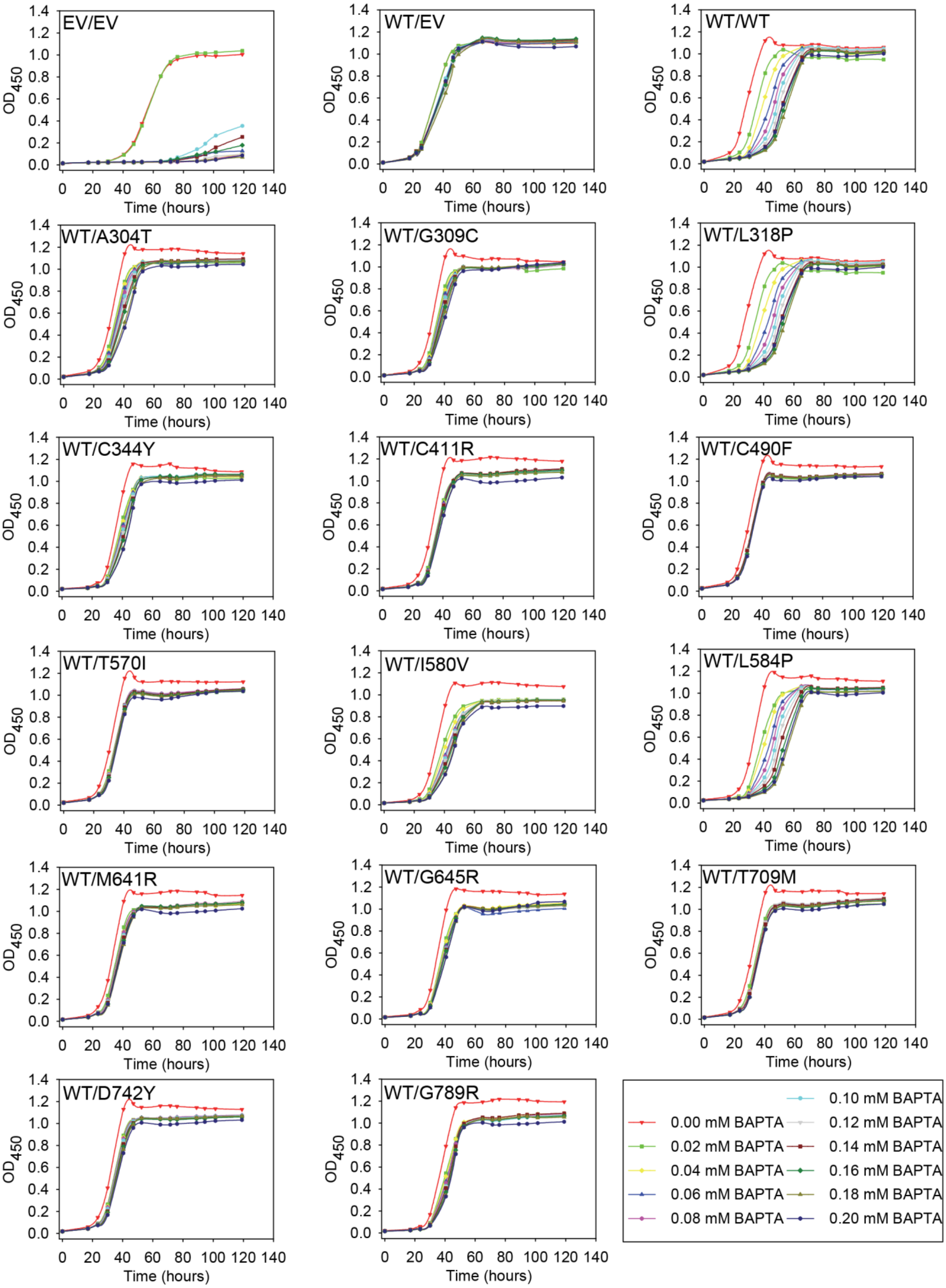
EV/EV
WT/EV
WT/WT
WT/A304T
WT/G309C
WT/L318P
WT/C344Y
WT/C411R
WT/C490F
WT/T570I
WT/I580V
WT/L584P
WT/M641R
WT/G645R
WT/T709M
WT/D742Y
WT/G789R
OD450
Time (hours)
0.00 mM BAPTA
0.02 mM BAPTA
0.04 mM BAPTA
0.06 mM BAPTA
0.08 mM BAPTA
0.10 mM BAPTA
0.12 mM BAPTA
0.14 mM BAPTA
0.16 mM BAPTA
0.18 mM BAPTA
0.20 mM BAPTA

Supplementary Figure S4. Complementation of the calcium dependent phenotype of the *pmr1*Δ *pmc1*Δ *cnb1*Δ yeast host strain by co-expression of wt-hSPCA1 and HHD-variants from integrative vectors.

Yeast strain PAP4920 expressing no hSPCA1a (Empty Vector, EV/EV) or co-expressing wt hSPCA1a (WT) from the integrative vector pPAP7010 with empty vector (WT/EV), wt hSPCA1a (WT/WT) or the indicated HHD hSPCA1a variants (WT/HHD-variant) from the integrative vector pPAP5480 (Supplementary Fig. S1) were grown at 30°C in 96-well plates in liquid media with galactose as carbon source supplemented with 20 mM $CaCl_2$ or BAPTA concentrations ranging from 0 to 0.2 mM in increments of 0.02 mM as indicated. Each culture was grown in triplicates and the average OD_{450} at the given time was plotted with respect to time of inoculation. Growth media containing BAPTA (including 0 mM BAPTA) do not have any added Ca^{2+}.

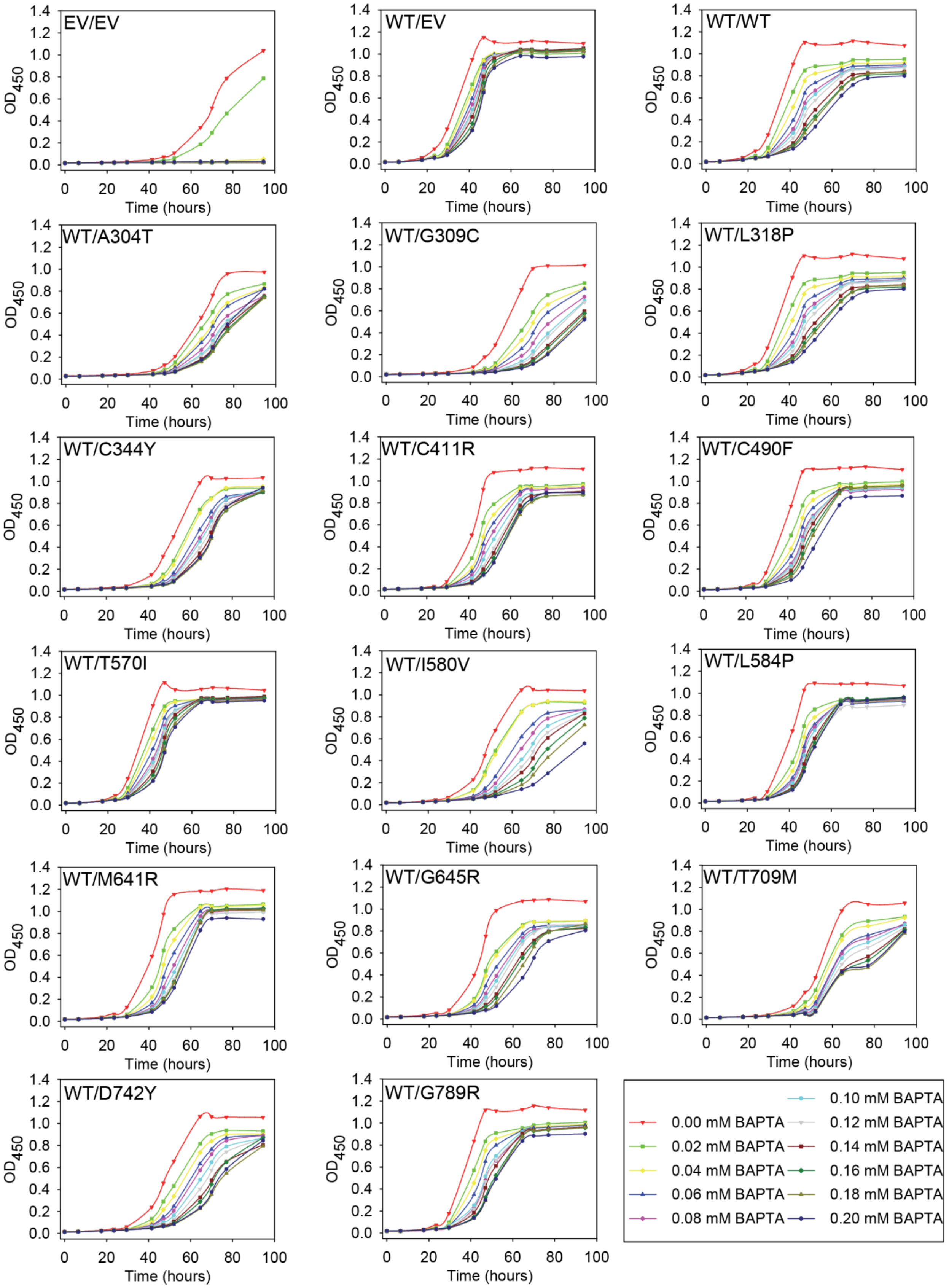
EV/EV
WT/EV
WT/WT
WT/A304T
WT/G309C
WT/L318P
WT/C344Y
WT/C411R
WT/C490F
WT/T570I
WT/I580V
WT/L584P
WT/M641R
WT/G645R
WT/T709M
WT/D742Y
WT/G789R
OD450
Time (hours)
0.00 mM BAPTA
0.02 mM BAPTA
0.04 mM BAPTA
0.06 mM BAPTA
0.08 mM BAPTA
0.10 mM BAPTA
0.12 mM BAPTA
0.14 mM BAPTA
0.16 mM BAPTA
0.18 mM BAPTA
0.20 mM BAPTA

Supplementary Figure S5. Complementation of the calcium dependent phenotype of the *pmr1*Δ *pmc1*Δ *cnb1*Δ yeast host strain by co-expression of wt-hSPCA1 and HHD-variants from replicative vectors.

Yeast strain PAP4920 expressing no hSPCA1a (Empty Vector, EV/EV) or co-expressing wt hSPCA1a (WT) from pPAP7177 with empty vector (WT/EV), wt hSPCA1a with wt hSPCA1a (WT/WT) or the indicated HHD hSPCA1a variants (WT/HHD-variant) from the replicative vector pPAP4997 (Supplementary Fig. S1) were grown at 30°C in 96-well plates in liquid media with galactose as carbon source supplemented with 20 mM $CaCl_2$ or BAPTA concentrations ranging from 0 to 0.2 mM in increments of 0.02 mM as indicated. Each culture was grown in triplicates and the average OD_{450} at the given time was plotted with respect to time of inoculation. Growth media containing BAPTA (including 0 mM BAPTA) do not have any added Ca^{2+}.

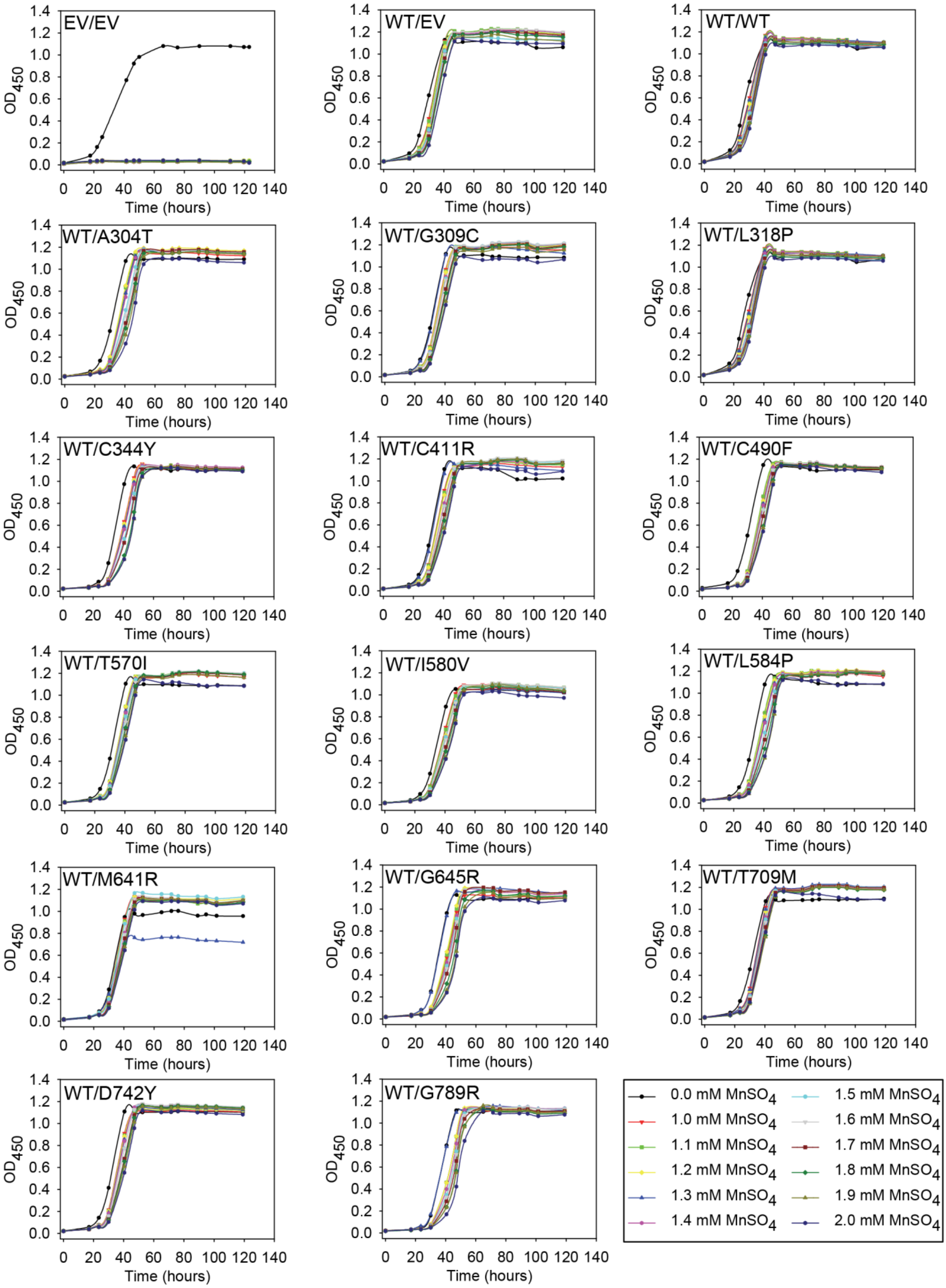

EV/EV
WT/EV
WT/WT
WT/A304T
WT/G309C
WT/L318P
WT/C344Y
WT/C411R
WT/C490F
WT/T570I
WT/I580V
WT/L584P
WT/M641R
WT/G645R
WT/T709M
WT/D742Y
WT/G789R
OD_{450}
Time (hours)
0.0 mM $MnSO_4$
1.0 mM $MnSO_4$
1.1 mM $MnSO_4$
1.2 mM $MnSO_4$
1.3 mM $MnSO_4$
1.4 mM $MnSO_4$
1.5 mM $MnSO_4$
1.6 mM $MnSO_4$
1.7 mM $MnSO_4$
1.8 mM $MnSO_4$
1.9 mM $MnSO_4$
2.0 mM $MnSO_4$

Supplementary Figure S6. Complementation of the manganese sensitive phenotype of the *pmr1*Δ *pmc1*Δ *cnb1*Δ yeast host strain by co-expression of wt-hSPCA1 and HHD-variants from integrative vectors.

Yeast strain PAP4920 expressing no hSPCA1a (Empty Vector, EV/EV) or co-expressing wt hSPCA1a (WT) from the integrative vector pPAP7010 with empty vector (WT/EV), wt hSPCA1a (WT/WT) or the indicated HHD hSPCA1a variants (WT/HHD-variant) from the integrative vector pPAP5480 (Supplementary Fig. S1) were grown at 30°C in 96-well plates in liquid media with galactose as carbon source supplemented with 20 mM $CaCl_2$, and $MnSO_4$ ranging from 0 to 2.0 mM in increments of 0.1 mM as indicated. Each culture was grown at 30°C, in triplicates, and average OD_{450} was plotted with respect to time of inoculation.

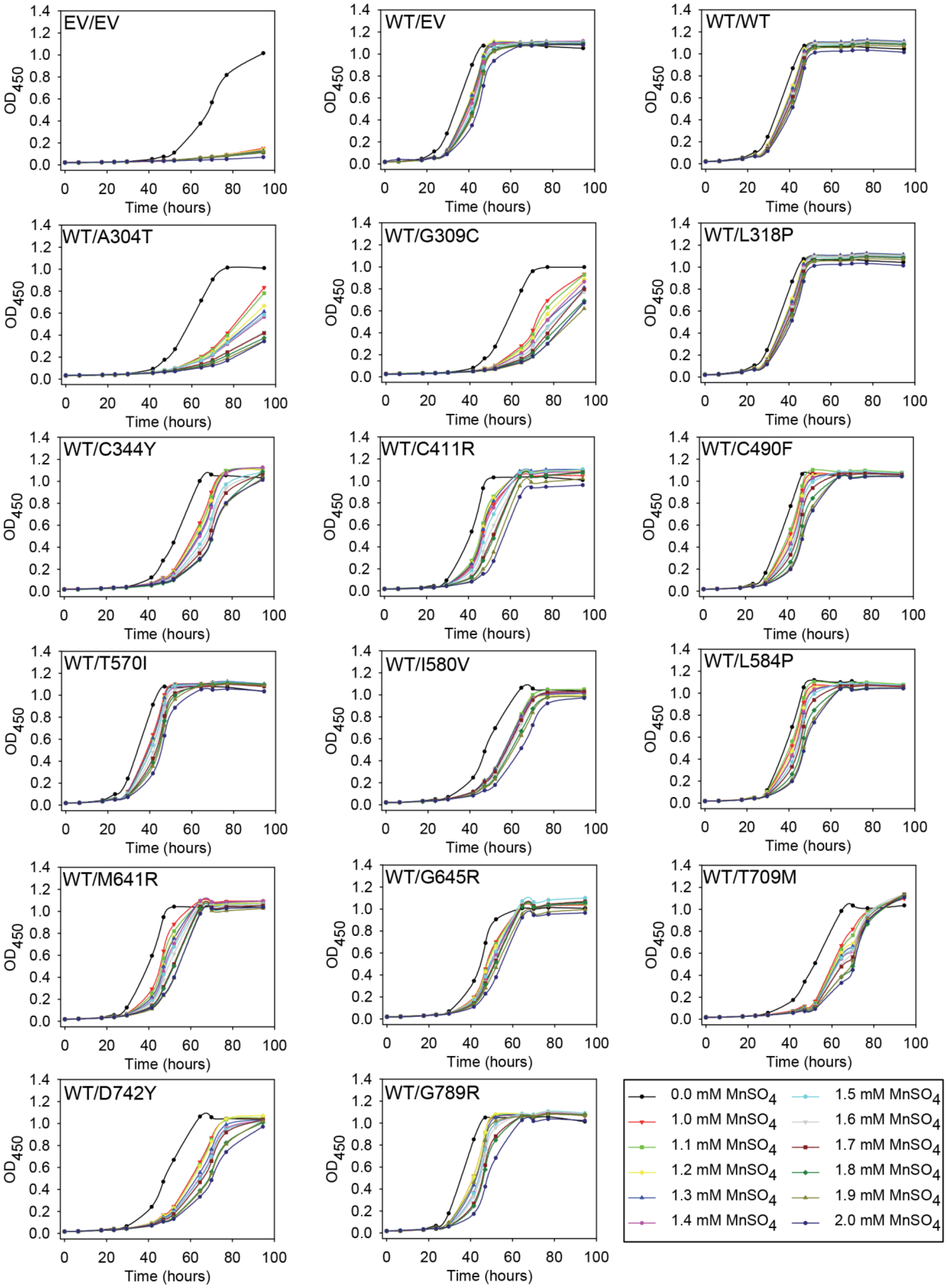
EV/EV
WT/EV
WT/WT
WT/A304T
WT/G309C
WT/L318P
WT/C344Y
WT/C411R
WT/C490F
WT/T570I
WT/I580V
WT/L584P
WT/M641R
WT/G645R
WT/T709M
WT/D742Y
WT/G789R
OD450
Time (hours)
0.0 mM MnSO4
1.0 mM MnSO4
1.1 mM MnSO4
1.2 mM MnSO4
1.3 mM MnSO4
1.4 mM MnSO4
1.5 mM MnSO4
1.6 mM MnSO4
1.7 mM MnSO4
1.8 mM MnSO4
1.9 mM MnSO4
2.0 mM MnSO4

Supplementary Figure S7. Complementation of the manganese sensitive phenotype of the *pmr1*Δ *pmc1*Δ *cnb1*Δ yeast host strain by co-expression of wt-hSPCA1 and HHD-variants from integrative vectors.

Yeast strain PAP4920 expressing no hSPCA1a (Empty Vector, EV/EV) or co-expressing wt hSPCA1a (WT) from pPAP7177 with empty vector (WT/EV), wt hSPCA1a with wt hSPCA1a (WT/WT) or the indicated HHD hSPCA1a variants (WT/HHD-variant) from the replicative vector pPAP4997 (Supplementary Fig. S1) were grown at 30°C in 96-well plates in liquid media with galactose as carbon source supplemented with 20 mM $CaCl_2$, and $MnSO_4$ ranging from 0 to 2.0 mM in increments of 0.1 mM as indicated. Each culture was grown at 30°C, in triplicates, and average OD_{450} was plotted with respect to time of inoculation.

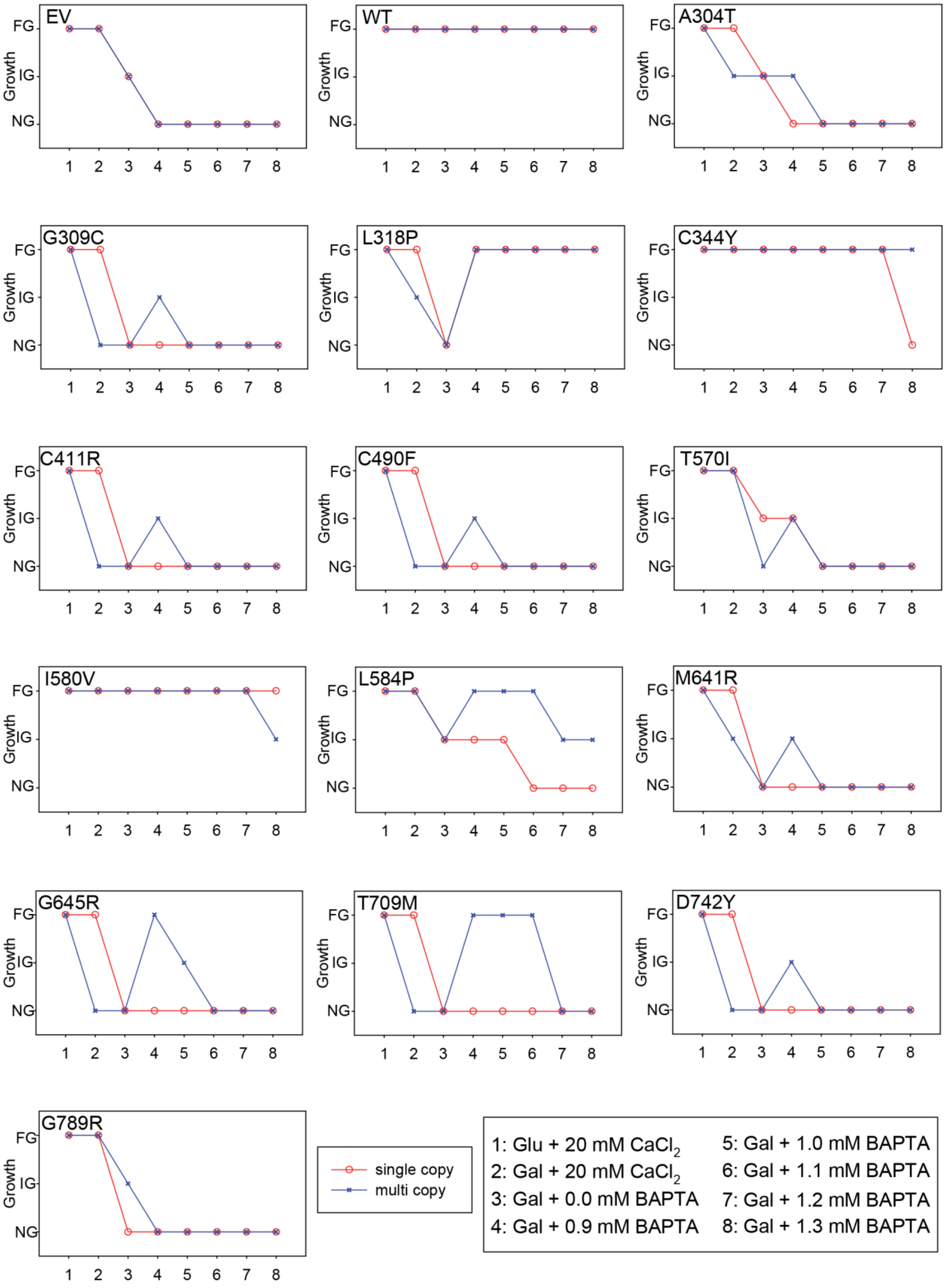
EV
WT
A304T
G309C
L318P
C344Y
C411R
C490F
T570I
I580V
L584P
M641R
G645R
T709M
D742Y
G789R
Growth
FG
IG
NG
1 2 3 4 5 6 7 8
single copy
multi copy
1: Glu + 20 mM $CaCl_2$
2: Gal + 20 mM $CaCl_2$
3: Gal + 0.0 mM BAPTA
4: Gal + 0.9 mM BAPTA
5: Gal + 1.0 mM BAPTA
6: Gal + 1.1 mM BAPTA
7: Gal + 1.2 mM BAPTA
8: Gal + 1.3 mM BAPTA

Supplementary Figure S8. Ca^{2+} sensitive growth phenotype.

Growth of the spots in Fig. 6 at 30°C was categorized as either wt-like (Full Growth, FG), intermediate (Intermediate Growth, IG) or no growth (NG) and plotted for each of the media. Red curves represent expression from the integrative vector pPAP5480 and blue curves represent expression from the replicative vector pPAP4997. A more or less linear correlation between Ca^{2+} availability and growth is observed for the empty vector (EV). Several of the investigated HHD mutants display a non-linear correlation between Ca^{2+} availability and growth, by growing very poorly or not being able to grow at all in media containing galactose and 20mM $CaCl_2$ or galactose and 0mM $CaCl_2$. This Ca^{2+} sensitive growth phenotype is observed as a "dip" in the curves in media containing either galactose and 20mM $CaCl_2$ or galactose and 0mM BAPTA (no $CaCl_2$ added) or both. Note that HHD-variants that display a Ca^{2+} sensitive growth phenotype are able to grow to some extent on media containing 0.9mM or higher BAPTA concentrations.

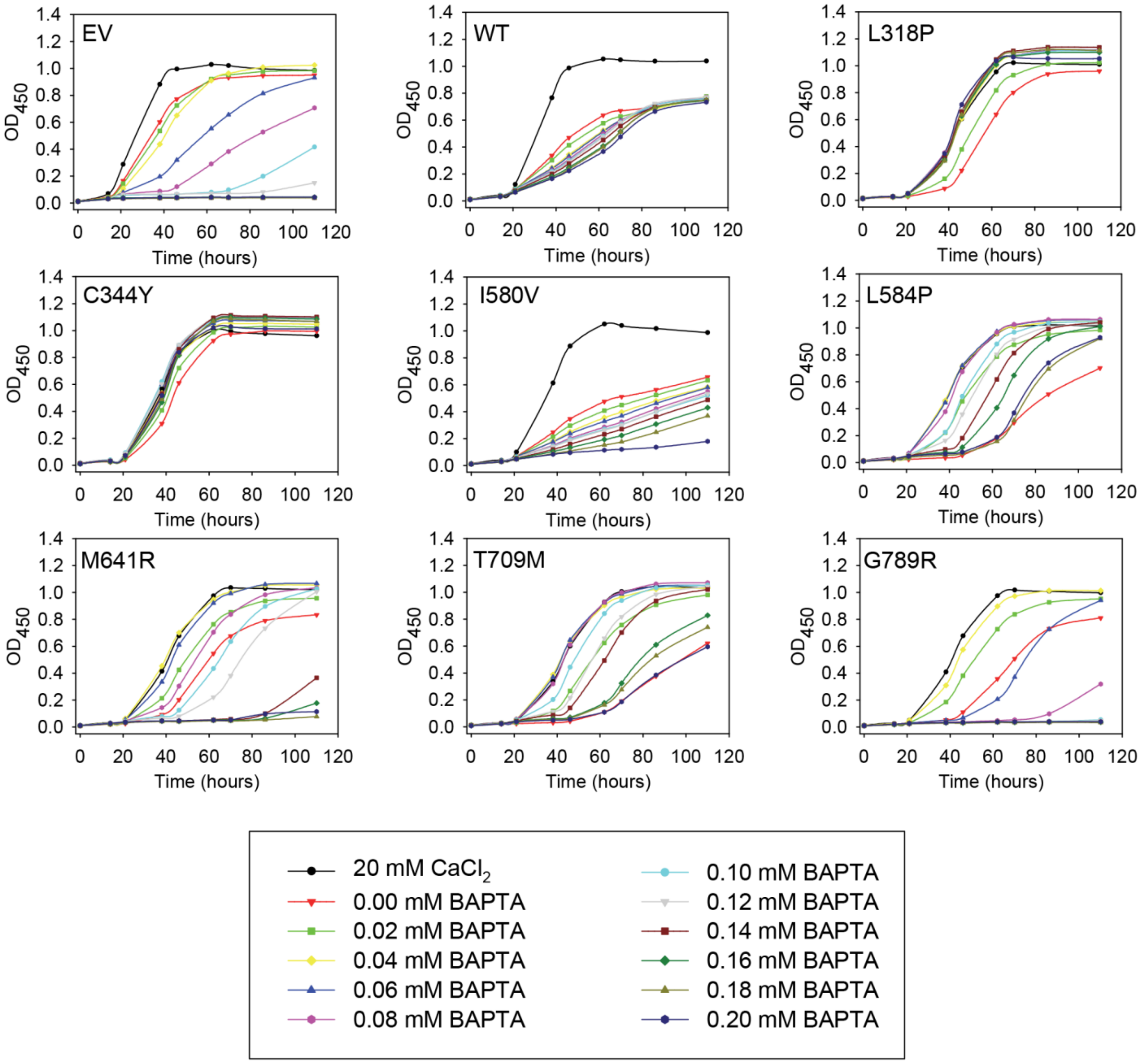

Supplementary Figure S9. Complementation from the integrative vector at 35°C confirms the temperature dependent complementation observed on agar plates at 35°C.

Yeast strain PAP4920 expressing no hSPCA1a (Empty Vector, EV), wt hSPCA1a (WT) or the indicated HHD hSPCA1a variants from the integrative vector (pPAP5480, Supplementary Fig. S1) were grown at 35°C in 96-well plates in liquid media containing galactose as carbon source

supplemented with 20 mM $CaCl_2$ or BAPTA concentrations ranging from 0 to 0.2mM in increments of 0.02 mM as indicated. Each culture was grown in triplicates and the average OD_{450} at the given time was plotted with respect to time of inoculation.

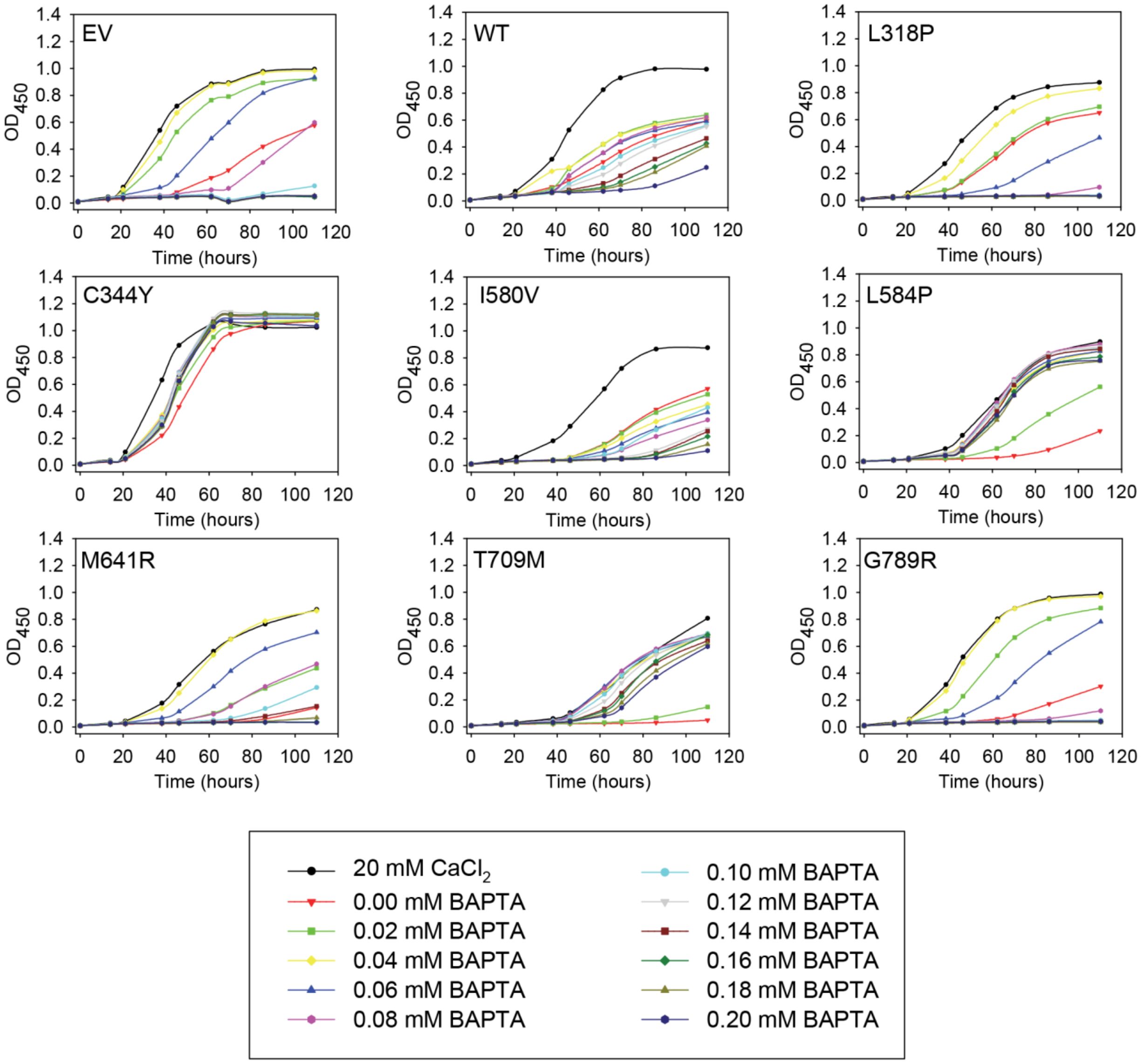

Supplementary Figure S10. Complementation from the replicative vector at 35°C confirms the temperature dependent complementation observed on agar plates at 35°C.

Yeast strain PAP4920 expressing no hSPCA1a (Empty Vector, EV), wt hSPCA1a (wt) or the indicated HHD hSPCA1a variants from the replicative vector (pPAP4997, Supplementary Fig. S1) were grown at 35°C in 96-well plates in liquid media containing galactose as carbon source supplemented with

20 mM $CaCl_2$ or BAPTA concentrations ranging from 0 to 0.2mM in increments of 0.02 mM as indicated. Each culture was grown in triplicates and the average OD_{450} at the given time was plotted with respect to time of inoculation.

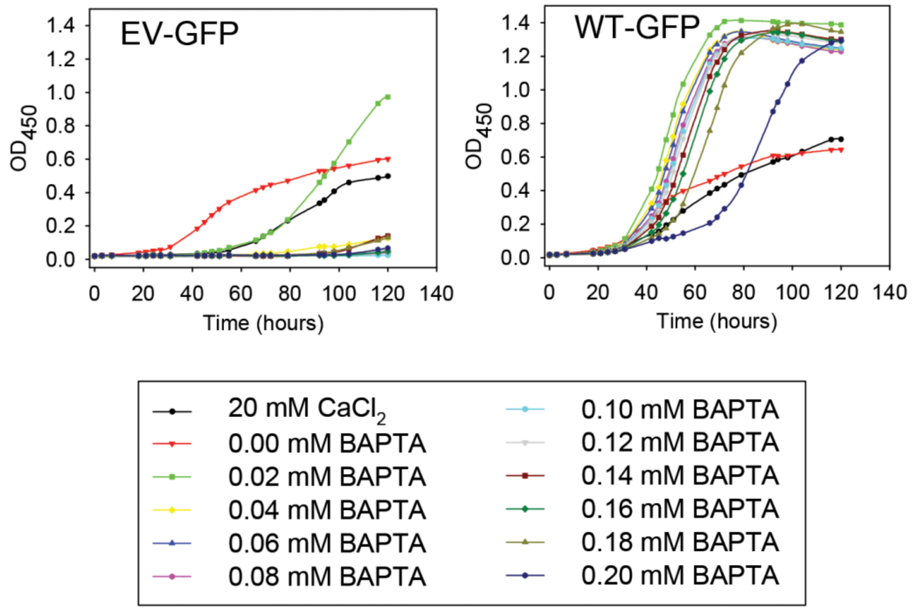

Supplementary Figure S11. Fusion of GFP to the C-terminus of wt hSPCA1a does not alter the complementation capacity of the un-tagged wt hSPCA1a. (See Fig. 3). Growth of yeast strain PAP4920 carrying the replicative empty vector (EV) or the replicative vector encoding the wt hSPCA1a-GFP fusion in micro plates at 30°C in the indicated 12 different growth media. Each culture was grown in triplicates and the average OD_{450} at the given time was plotted with respect to time of inoculation.

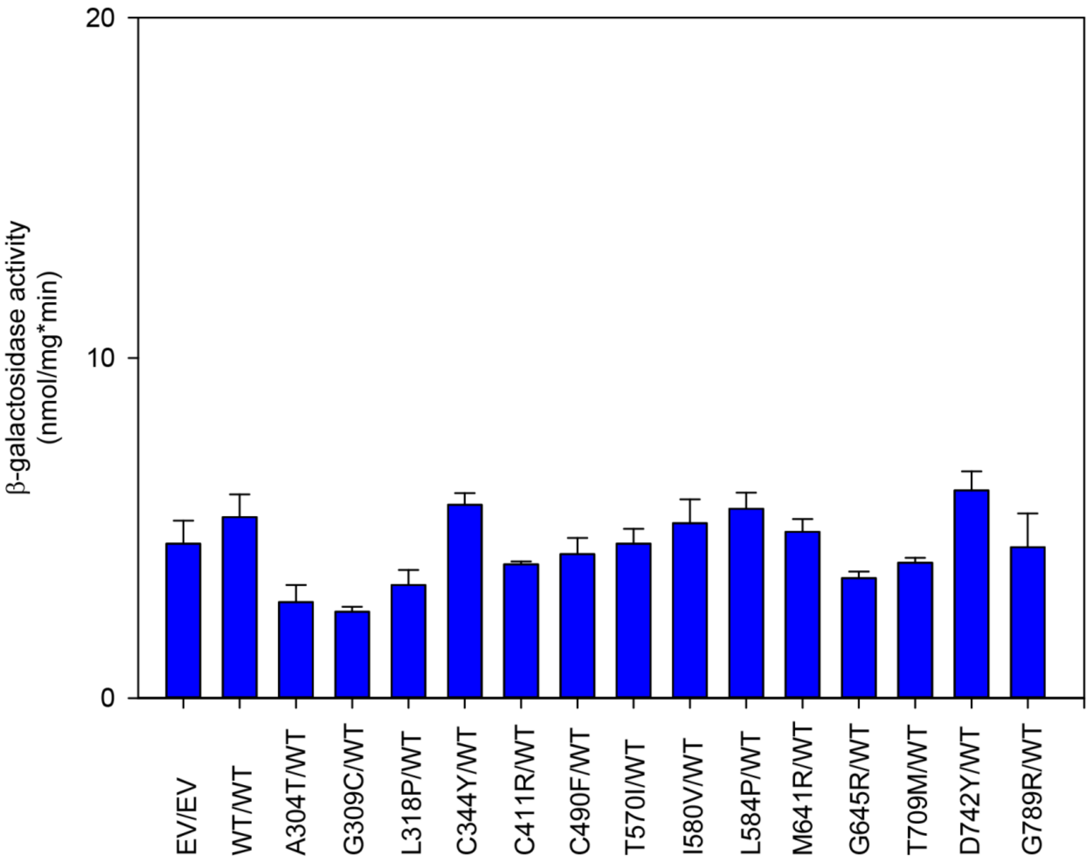

Supplementary Figure S12. Co-expression of wt-hSPCA1a with either of the investigated HHD-alleles induces a very low unfolded protein response (UPR). Yeast strain PAP9588 carries the chromosomal unfolded protein response reporter UPR-*lacZ* that can be utilized to monitor *in-vivo* protein folding. The cytosolic fractions from yeast strains expressing no ATPases (empty vector, EV/EV) or wt-hSPCA1a (WT) from the replicative vector pPAP8754 along with wt-hSPCA1a (WT/WT) or one of the 14 investigated HHD-variants from the replicative vector pPAP4997 (HHD/WT) were analyzed for β-galactosidase activity as described in Materials and Methods. Yeast cultures were inoculated in standard minimal medium supplemented with 20 mM $CaCl_2$ and

galactose as sole carbon source in shake flasks at 30°C at OD_{450} = 0.05. Cells were harvested when OD_{450} = 1 – 1.5. Results represent mean and standard deviations from three estimates.

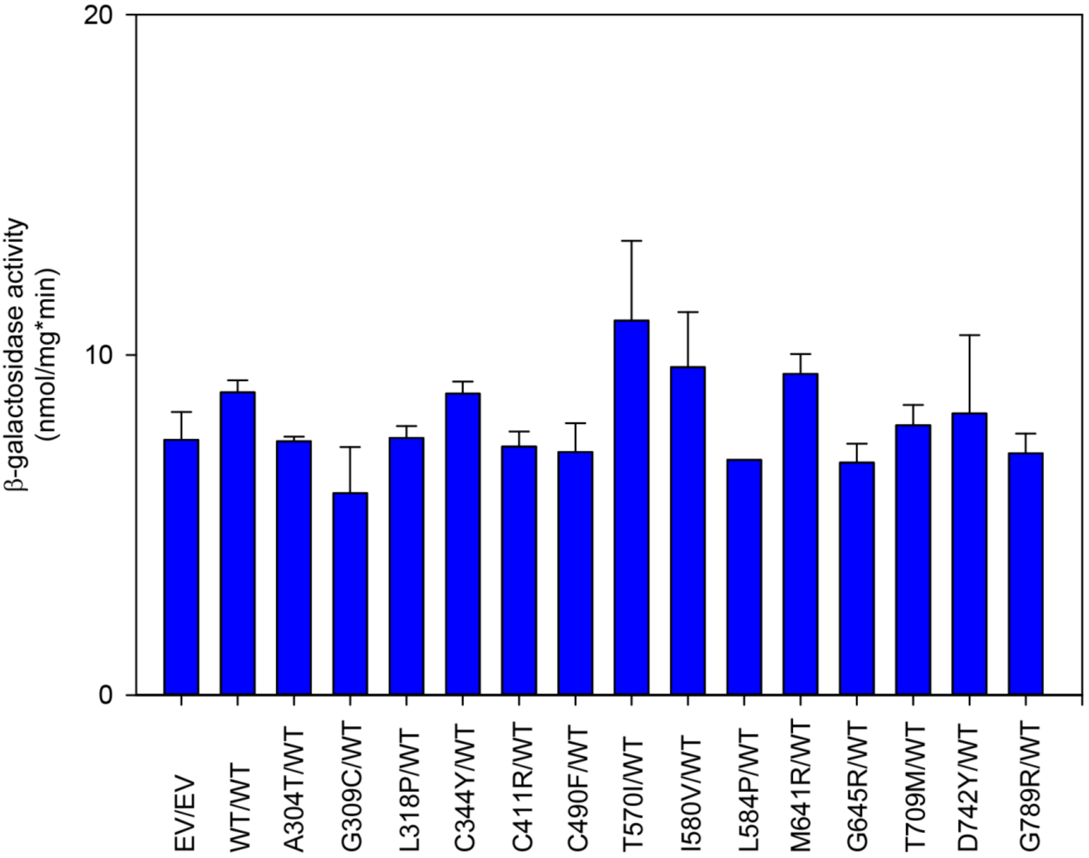

Supplementary Figure S13. Co-expression of wt-hSERCA2b with either of the investigated HHD-alleles induces a very low unfolded protein response (UPR). Yeast strain PAP9588 carries the chromosomal unfolded protein response reporter UPR-*lacZ* that can be utilized to monitor *in-vivo* protein folding. The cytosolic fractions from yeast strains expressing no ATPases (empty vector, EV/EV) or wt-hSERCA2b (WT) from the replicative vector pPAP9588 along with wt-hSPCA1a (WT/WT) or one of the 14 investigated HHD-variants from the replicative vector pPAP4997 (HHD/WT) were analyzed for β-galactosidase activity as described in Materials and Methods. Yeast cultures were inoculated in standard minimal medium supplemented with 20 mM $CaCl_2$ and

galactose as sole carbon source in shake flasks at 30°C at OD_{450} = 0.05. Cells were harvested when OD_{450} = 1 – 1.5. Results represent mean and standard deviations from three estimates.

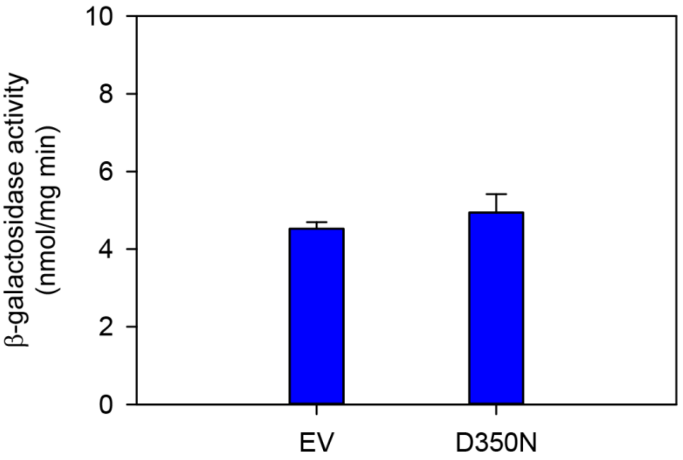

Supplementary Figure S14. Expression of the enzymatically dead D350N mutant in yeast strain PAP4920 induces a very low unfolded protein response (UPR).

Yeast strain PAP4920 carries the chromosomal unfolded protein response reporter UPR-*lacZ* that can be utilized to monitor *in-vivo* protein folding. The cytosolic fractions from yeast strains expressing no ATPase (empty vector, EV) or the enzymatically dead D350N variant from the replicative vector pPAP4997 were analyzed for β-galactosidase activity as described in Materials and Methods. Yeast cultures were inoculated in standard minimal medium supplemented with 20 mM $CaCl_2$ and galactose as sole carbon source in shake flasks at 30°C at OD_{450} = 0.05. Cells were harvested when OD_{450} = 1 – 1.5. Results represent mean and standard deviations from three estimates.

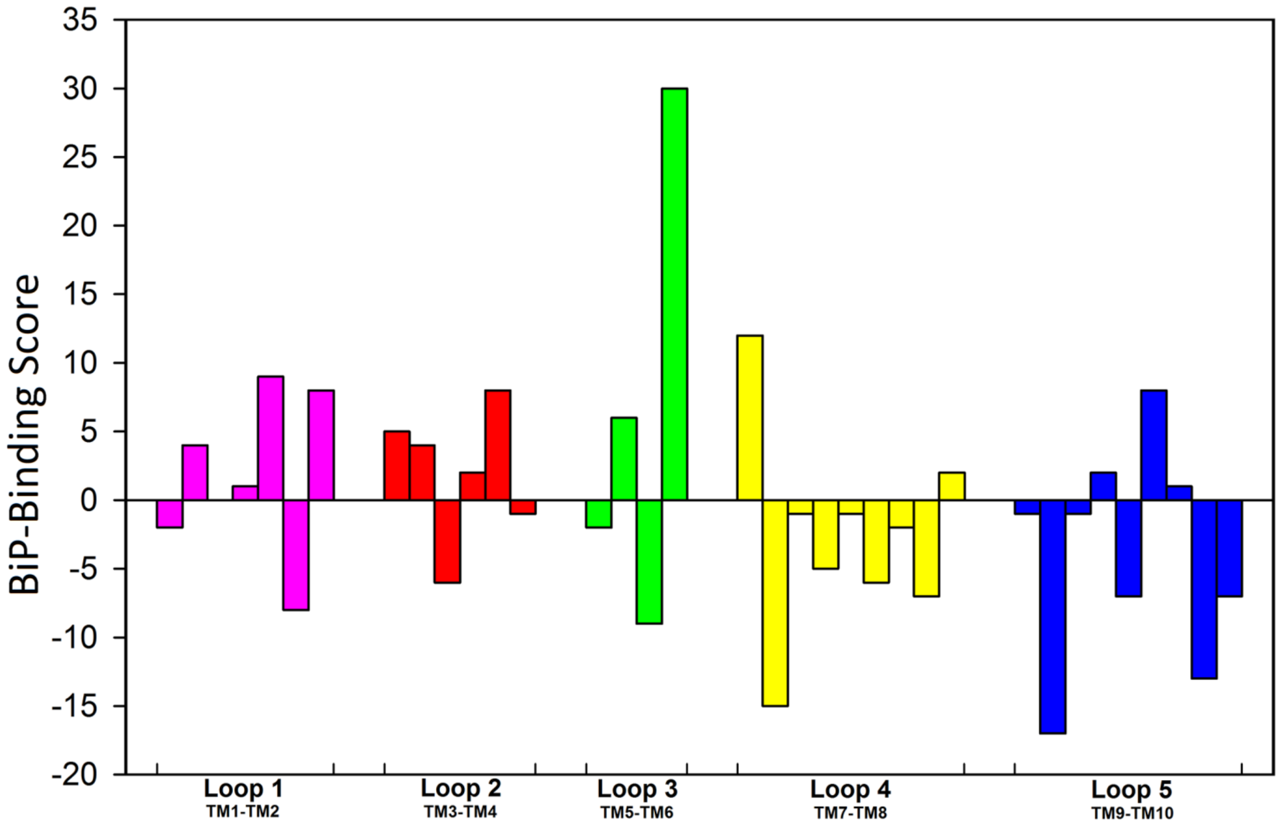

Supplementary Figure S15. Bioinformatics predicts two BiP binding sites in hSPCA1a.

The 919 amino acids long hSPCA1a protein only exposes the 47 amino acids connecting the ten transmembrane segments to the ER lumen. We therefore screened the primary structure of these loops for presence of a potential BiP binding site using an algorithm developed by Blond-Elguindi et al.[100]. Based on the predicted secondary structure in Fig. 1, a score for each possible hepta-peptide located in each of the five ER loops (indicated by different color) was calculated. Hepta-petides with scores greater than 10 have very high probability for binding to BiP, while scores between 6 and 10 have odds 3:1 for binding BiP. Scores between 0 and 5 have very low probability of BiP binding, while negative scores predict that the hepta-peptide is unable to bind BiP. Loop 3 (located between TM5 and TM6, indicated by green color) contains the most probable BiP binding site (723-MNFPNPL-729) with a BiP score of +30, well above the threshold value of +10 that

indicates an extremely high probability for BiP binding. Two hepta-peptides (amino acids 96 to 102 and 98 to 104 respectively) located in the TM1-TM2 loop score +9 and +8, respectively and a single hepta-peptide sequence located in TM9-TM10 (amino acid 866 to 872) has a BiP score of +8 all indicating a 3:1 probability of BiP binding. Two other hepta-peptides in luminal loop 5 each scores +4 which according to Blond-Elguindi et al., is of little predictive value.

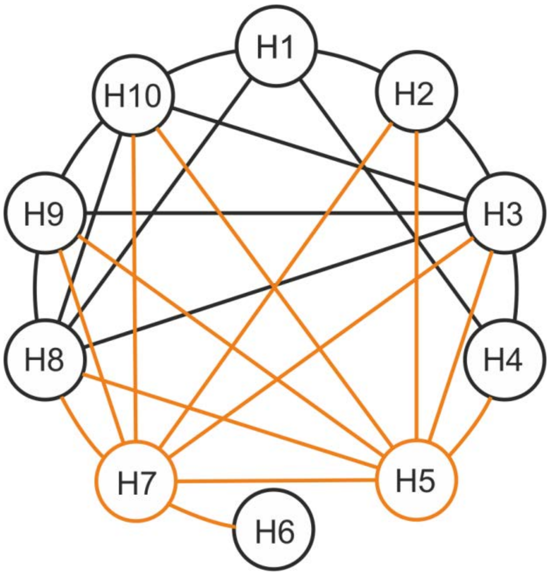

Supplementary Figure S16. Predicted helix-helix interactions in the TM-region of hSPCA1a. We used the TMhit tool[102] to predict interactions among the ten trans-membrane helices in hSPCA1a. Numbers identify the number of individual helices in the hSPCA1a primary structure (Fig. 1). Line connecting helixes depicts a possible helix-helix interaction. Interactions involving helix 5 and helix 7 are indicated in orange.
